# Neural Expression of IL-16 Mediates Neuropathic Pain after Sciatic Injury in Mice

**DOI:** 10.1007/s12035-026-06280-2

**Published:** 2026-10-22

**Authors:** Alba Coro-Martín, Alejandro Álvarez-Artime, Sara González-Rodríguez, María González-Amor, David Corral-Fontecha, Ana Baamonde, Luis Menéndez

**Affiliations:** 1 Farmacología, Facultad de Medicina, Instituto Universitario de Oncología del Principado de Asturias (IUOPA), Universidad de Oviedo, C/Julián Clavería 6, Oviedo, Asturias 33006 Spain; 2 Instituto de Investigación Sanitaria del Principado de Asturias (ISPA), Oviedo, Spain; 3 Departamento de Radiología, Complejo Asistencial Universitario de León, León, Spain

**Keywords:** Neuropathic pain, IL-16, Sciatic nerve, Hyperalgesia, Allodynia, CD4 receptors

## Abstract

The neutralization of interleukin-16 (IL-16) or of its main target, CD4 receptors, as well as the reduction of its synthesis by inhibiting caspase-3 have been recently considered as possible strategies to inhibit pain. Here we study the involvement of peripheral mechanisms related to the IL-16/CD4 axis in the nociceptive responses evoked after ligation of sciatic nerve in mice. Systemic administration of anti-IL-16 or anti-CD4 antibodies inhibited hyperalgesia and allodynia measured 2 days after ligation in male and female mice. In addition, both antibodies were also analgesic 9 days after nerve ligation, supporting the participation of IL-16 and CD4 in the establishment and maintenance of neuropathic pain. Following nerve injury, *Il16* expression remained unaltered in dorsal root ganglia (DRGs) and damaged nerves, whereas *Casp3* expression increased in DRGs and the enzymatic activity of caspase-3 was enhanced in damaged nerves. Immunofluorescence assays demonstrated a limited presence of IL-16 in DRG neurons, that remained unmodified after injury, and a clear up-regulation of this cytokine in injured nerves. Double immunofluorescence assays with βIII-tubulin and S100β antibodies demonstrated that IL-16 is expressed both in axons and Schwann cells after nerve ligation. The ability of IL-16 to evoke nociception by directly acting on nerves was confirmed by intra-sciatic administration. Immunofluorescence labelling of CD4 receptors was also present in injured nerves, being also up-regulated both in axons and Schwann cells. Finally, hyperalgesia and allodynia were prevented by the intra-sciatic administration of anti-IL-16 or anti-CD4 antibodies, thus supporting the relevance of the IL-16/CD4 axis in neuropathic hypernociception.

**Supplementary Information:**

The online version contains supplementary material available at https://doi.org/10.1007/s12035-026-06280-2.

## Introduction

Several interleukins are involved in the modulation of nociceptive transmission and, although some particular examples, as IL-10 [1], can provoke analgesic effects, the vast majority are involved in inflammatory reactions promoting the sensitization of peripheral and central nociceptive neurons and contributing to pain amplification. Thus, inflammatory interleukins, such as IL-1β, IL-6, IL-17, IL-18 and IL-33 are up-regulated in the periphery and the spinal cord in different inflammatory [2], neuropathic [3] or neoplastic painful settings [4] and the inhibition of their action can be considered a useful analgesic strategy [2]. Among pro-inflammatory cytokines, interleukin-16 (IL-16), formerly known as lymphocyte chemoattractant factor, has started to be envisaged as a potential therapeutic target based on recent studies focussed on its involvement in nociceptive transmission modulation [5].

The administration of exogenous IL-16 to mice at peripheral level (intraplantar or perineural) evokes nociceptive reactions in laboratory animals [6–8] through the stimulation of CD4 receptors [7]. The activation of both COX-1 and COX-2 as well as TRPV1 or TRPA1 channel sensitization seem to participate in these effects [7]. Besides, the direct intrathecal injection of this cytokine also evokes thermal hyperalgesia and mechanical allodynia reactions [8, 9] which are related to CD4 receptor stimulation and are accompanied by astroglial and T cell activation [9]. Furthermore, the possible role of IL-16 in different pathological models of nociception has been also explored by the direct inhibition of IL-16 or by preventing its synthesis. In animal models of peripheral inflammatory pain, it has been shown that neutralization of IL-16 or CD4 receptors by antibodies systemically administered effectively reduces both thermal hyperalgesia and mechanical allodynia [7]. Similarly, the knockdown of IL-16 by the intrathecal injection of IL-16 siRNA is another useful strategy to attenuate these pain symptoms [10]. Also, the neutralization of IL-16 or CD4 receptors has proven useful to counteract painful reactions due to the intraplantar or intraarticular inoculation of *Staphylococcus aureus* in mice [11]. In all these models, enhanced concentrations of IL-16 appear in response to the tissue insult either peripherally [7, 11] or into the spinal cord [10].

The relevance of IL-16 after experimental neuropathic injury has been specially focussed on the molecular changes that occur into the spinal cord. Thus, in mice bearing neuropathic pain after spinal nerve ligation, an increased presence of IL-16^+ ^and CD4^+^ cells in the spinal dorsal horn as well as the up-regulation of *Il16* mRNA has been reported. Moreover, the reduction of mechanical and thermal neuropathic hypersensitivity either by the spinal IL-16 knockdown with siRNA or the inhibition of CD4 receptors has also been shown [9]. More recent data obtained in rats with neuropathic pain after chronic constriction of the sciatic nerve demonstrated IL-16 up-regulation at spinal cord, DRG and sciatic nerve of rats with neuropathy [8]. Whereas in this setting central IL-16^+^ cells correspond predominantly to neurons at the spinal cord, macrophages were suggested to be the main source of peripheral IL-16 after nerve injury [8].

The initial aim of this set of experiments was to explore the functional involvement of the IL-16/CD4 axis in the peripheral nociceptive processing during the early phases of the establishment of neuropathic painful symptoms in a commonly used experimental model in mice based in the triple ligature of the sciatic nerve. Behavioural assays were also addressed to assess possible gender differences and to explore whether the involvement of IL-16 could be exclusively related to the initial period after injury or be also operating during the long-term maintenance of this type of neuropathic pain, as suggested in previous reports [8, 9]. Furthermore, considering the recent description of the increased presence of IL-16 in nerves of rats submitted to ligation [8], we performed immunohistochemical assays to elucidate whether the possible increase in sciatic IL-16 expression could be related to axons, glial Schwann cells or immune cells infiltrated in response to injury, and also to assess the expression of CD4 receptors at this level. Since IL-16 synthesis is related to the activation of caspase-3, the expression and the activity of this enzyme was also studied in DRGs and sciatic nerves. Finally, in order to confirm in behavioural assays the ability of the IL-16/CD4 axis to amplify nociceptive transmission at peripheral nerves, the effect of IL-16 and anti-IL-16 or anti-CD4 antibodies was assessed after their direct intra-sciatic administration.

## Material and Methods

### Animals

Swiss CD-1 male mice 7–10-week-old bred in the Bioterio de la Universidad de Oviedo (Reg. 33,044 13 A) maintained on a 12-h dark–light cycle with food and water ad libitum were used and some key behavioural assays have been also performed in female mice. Experiments were done during the light cycle in a quiet room with controlled temperature (21 °C) and efforts were made to limit animal stress.

### Sciatic Nerve Ligation

Following the procedure previously described [12], mice were initially anesthetized by spontaneous inhalation of 3% isoflurane (Isoflo®, Ecuphar) in oxygen through a breathing mask. After shaving the surgical field, 10% povidone-iodine (Montplet Laboratorios) was applied and surgical instrumentation was sterilized (MicroBead Sterilizer, Fisherbrand B1305-E-FIS) before use to perform the procedure aseptically. An incision was made at the skin of the right mid-thigh parallel to femur and at the biceps femoris muscle, the sciatic nerve was exposed and 3 ligatures with 6/0 silk were placed around the nerve at 3-mm distance between them. The muscular layer of the biceps femoris was closed with absorbable suture and the skin was closed with 3/0 silk and acrylic glue (Hystoacril®, Braun). Sham procedure was performed in a similar manner but without nerve ligation. Experiments were performed 2 days after surgery, except in a particular behavioural assay carried out at day 9. Mice were sacrificed after the experiment.

### Drugs

Antibodies against mouse IL-16 (R&D, MAB1727) or CD4 (clone RM4-4, Stem Cell 60,029) were initially reconstituted in PBS and next dissolved in saline for their subcutaneous administration. Control mice received an injection of the same volume of the corresponding solvent or IgG isotype (IgG2bк, eBioscience 14–4031-85). Mouse recombinant IL-16 (Raybiotech, 230–30050-10) was dissolved in saline.

The intraperitoneal (i.p.) administration 24 h before testing of an anti-CD3 antibody (1 μg/300 µl; eBioscience 14–0032–82) diluted in saline was used to evoke lymphocyte depletion, as previously described [13, 14]. For neutrophil-depletion experiments, mice were injected intraperitoneally with the anti-Ly6G antibody (BioXcell) (100 µg dissolved in 100 µl of Phosphate Buffered Saline) [13, 15]. Subcutaneous (s.c.) administration was performed under the fur of the neck in a volume of 10 ml/kg. Intra-sciatic (i.sc.) administration was performed following a previously described procedure [16] with slight modifications. In mice under 2% isoflurane anaesthesia (Isoflo®, Ecuphar), the right sciatic nerve was exposed and held by using an open surgical forceps with a maximal opening segment of about 6 mm to avoid excessive stretching. Nerve was visualized at a final 40 × magnification with a stereomicroscope. A borosilicate glass pipette (Drummond Scientific Company) was stretched with a gravity puller (LIST-Medical Electronic) to obtain a needle micropipette with 3 MΩ of resistance and filled afterwards with drug or solvent. By using a micromanipulator (Narishigue 11,845), the tip of the micropipette was carefully introduced inside the nerve at a < 45° acute angle by puncturing the epineurium with the tip of the micropipette oriented following the direction of the nerve towards the limb. Although the introduction of the tip sometimes occurred at first attempt, it was frequently necessary to advance and withdraw the glass pipette slightly (generally no more than 4–5 times) until the opening of a small window into the epineurium that allowed the introduction of the tip a distance of 0.5–1 mm approximately. Solutions were injected by using a pump (Syringe infusion pump 22, Harvard Apparatus) at a flow of 20 µl/h during 15 min, thus injecting a total volume of 5 µl. During the procedure, care was taken to keep the exposed nerve moistened by gently flushing saline solution over it. Initially, in order to check if this procedure could lead to an exclusive local action on sciatic nerve, the diffusion of methylene blue (0.1%; Merck) along the nerve was monitored. After drug administration, the nerve was relocated in its place, muscle layers were sutured in apposition and the skin was closed with a surgical staple. Once anaesthesia was over, mice showed no gait disturbances and were studied either 1 h or 2 days after the procedure.

### Unilateral Hot Plate

This test was used to detect the presence of thermal hyperalgesia in mice. As previously described [17], mice were gently restrained by the fur of the neck and the plantar side of each paw placed on a hot plate (IITC Life Science) set at 50 °C. This procedure allows to obtain separate measures of hind paws. Withdrawal latencies from the heated surface of each hind paw was done twice, at two minute-intervals, and the mean value was calculated. For habituation to the procedure, a basal measurement was done in each paw the morning of the experimental day (data not shown) and testing was performed during the afternoon. Cut-off was 20 s.

### Von Frey Test

The application of von Frey filaments to the plantar side of the hind paws allows to detect the presence of mechanical allodynia. Following the procedure previously used [11] mice were placed on the wire mesh platform and allowed to habituate for 60 min. Next, several von Frey filaments (Bioseb) were selected (2.44, 2.83, 3.22, 3.61, 4.08, 4.56, corresponding to a range of bending forces from 0.028 to 3.63 g). The procedure started with the 3.61 filament and six measurements were taken in each animal randomly starting with the left or right paw based on the ‘‘up and down’’ method [18]. A positive response consisting in lifting, shaking, licking of the paw or marked toe spreading was followed by the application of the immediate thinner filament. A negative response entailed the application of the immediate thicker filament. The following formula was used to calculate the 50% g threshold: 50% response threshold = (10Xf + κδ)/10,000. Xf is the value of the last von Frey filament applied; κ is a correction factor based on pattern of responses (from the Dixon’s calibration table); δ is the mean distance in log units between stimuli (here, 0.4).

### Immunofluorescence Experiments

Immunofluorescence assays were performed in dorsal root ganglia (DRGs) and sciatic nerves. When harvesting ligated nerves, knots were carefully dissected off trying to preserve intact the very thin constriction areas of nerves. All samples were fixed in 4% buffered paraformaldehyde (PFA, Labbox, FORM-P0P-10 K) at 4 °C. DRGs were maintained in PFA for 2 h, while sciatic nerves were immersed in PFA for 6 h. After fixation, DRGs and sciatic nerves were transferred to 15% sucrose (Sigma-Aldrich, Merck 84,100) for 6 h and 30% sucrose until cryosectioning up to a maximum of 3 days at 4 °C. Tissue sections of 16-µm thickness were obtained using a sliding microtome (Microm HM430) equipped with a rapid freezing unit KS34, by using 30% sucrose as cryoprotective medium.

Sections obtained in 30% sucrose did not readily adhere directly to the slides and they were collected and mounted manually. DRG and longitudinal sciatic nerve sections were collected using a fine single-bristle brush whereas the more delicate transversal sections of sciatic nerve were carefully aspirated from the blade using a pipette. Next, sections were transferred to a small drop of water placed on a Superfrost Plus™ microscope slide (VWR, 631–0108). The sections were then carefully unfolded, and extended manually using a fine single-bristle brush to preserve tissue integrity and the sections were allowed to air-dry at room temperature (RT).

In a particular experiment, thymus samples were collected and fixed 24 h in 4% PFA at 4 °C. After fixation, samples were immersed in 15% sucrose for 24 h and subsequently in 30% sucrose for at least 24 h. Then, 30-µm-thick tissue sections were obtained and mounted on Superfrost Plus™ microscope slides following the same protocol as before. These samples were always used fresh.

Prior to immunofluorescence assays, frozen slides were thawed and air-dried at room temperature (RT). DRG samples were permeabilized with 0.15% Tween-20 in PBS and sciatic nerve sections with 0.15% Triton X-100, in both cases for 20 min at RT. Next, they were blocked with 1.5% normal goat serum (Gibco, 1,153,026) in PBS for 1 h at RT. Subsequently, samples were incubated overnight at 4 °C in a humidified chamber with either a rabbit polyclonal anti-IL-16 antibody (1:100; Invitrogen PA5-82,627), a rabbit recombinant monoclonal anti-CD4 antibody (1:200; Proteintech 86,300–3-RR). In each case, one sample was maintained overnight in goat serum without primary antibody as a negative control (supplementary file 1). The following day, sections were washed three times with PBS and incubated for 1 h at RT with the secondary antibody Alexa Fluor 488 goat anti-rabbit (1:250; Invitrogen A-11008). After incubation, tissues were washed three times with PBS and counterstained with 1 µg/mL DAPI [2-(4-aminophenyl)−1H-indole-6-carboxamidine] (Invitrogen) for 10 min at RT. Sections were washed again three times with PBS and mounted using Fluoromount™ mounting medium (Sigma-Aldrich, Merck).

For CD3 assays, thymus and nerve sections were permeabilized with 0.15% Triton X-100 in PBS for 20 min at RT, and blocked with 5% bovine serum albumin (BSA; GoldBio, A-420–10) in PBS for 1 h at RT. Next, sections were incubated for 1 h at RT in a humidified chamber with a rat monoclonal anti-CD3 antibody (1:50; R&D Systems, MAB4841). Negative-control sections were incubated in blocking solution without the primary antibody (supplementary file 1). Sections were subsequently washed once with PBS and incubated for 1 h at RT with Alexa Fluor 546-conjugated goat anti-rat secondary antibody (1:250; Invitrogen, A-11081). Sections were then washed once with PBS and counterstained with 1 µg/mL DAPI for 10 min at RT and finally, after washing with PBS, they were mounted using Fluoromount™ mounting medium (Sigma-Aldrich, Merck).

Double immunofluorescence assays were performed to detect the expression of IL-16 in small sized DRG neurons labelled with peripherin. Samples were permeabilized with 0.15% Tween-20 in PBS for 20 min at RT, and blocked with 1.5% of normal donkey serum (Sigma, D9663) for 1 h at RT. Next, they were incubated with the goat anti-peripherin antibody (1:200, Santa Cruz Biotechnology, sc-7604) for 1 h at RT, washed with PBS 3 times and incubated with Alexa Fluor 555 donkey anti-goat secondary antibody (1:250, Invitrogen, A-11055) for 1 h at RT. Afterwards, samples were washed with PBS 5 times and were incubated overnight at 4 °C in a humidified chamber with the rabbit polyclonal anti-IL-16 antibody (1:100). The following day, sections were washed 3 times with PBS and incubated for 1 h at RT with the secondary antibody Alexa Fluor 488 goat anti-rabbit (1:250). As in the other assays, sections were then washed with PBS and counterstained with 1 µg/mL DAPI before mounting them using Fluoromount™ mounting medium (Sigma-Aldrich, Merck).

For double immunofluorescence experiments performed in nerves, tissues were fixed in 4% PFA for 3 h at RT. In transverse sections of 20-µm-thick the colocalization of IL-16 and CD4 receptor in axons or Schwann cells of ligated nerves the axon marker βIII-tubulin and the Schwann cell marker S100β were studied. To assess IL-16 or CD4 receptor expression in axons and Schwann cells, transverse sections of ligated sciatic nerves permeabilized with 0.15% Triton X-100 in PBS for 20 min at RT, were used. To perform the double labelling with βIII-tubulin sections were blocked with 1.5% normal goat serum in PBS for 1 h at RT. They were incubated overnight at 4 °C in a humidified chamber with either the rabbit polyclonal anti-IL-16 antibody (1:100) or the recombinant rabbit monoclonal anti-CD4 antibody (1:200). For axonal identification, sections were washed with PBS and incubated for 1 h at RT with an Alexa Fluor 594-conjugated mouse monoclonal anti-βIII-tubulin antibody (1:100; Santa Cruz Biotechnology, sc-80005) together with Alexa Fluor 488-conjugated goat anti-rabbit IgG (1:250) for the detection of either IL-16 or CD4. For the specific identification of Schwann cells, sections incubated as before with the anti-IL-16 (1:100) or anti-CD4 (1:200) antibodies were washed with PBS and incubated for 1 h at RT with the Alexa Fluor 488-conjugated goat anti-rabbit IgG (1:250). After washing with PBS, sections were blocked with 3% BSA for 1 h at RT. Next, they were incubated 1 h at RT with the Alexa Fluor 594-conjugated mouse monoclonal anti-S100β antibody (1:20; Santa Cruz Biotechnology, sc-393919). Finally, sections were washed with PBS, counterstained with 1 µg/mL DAPI for 10 min at RT, washed again with PBS, and mounted using Fluoromount™ mounting medium.

To assess IL-16 expression in neutrophils, longitudinal sections of ligated sciatic nerves were permeabilized with Triton X-100 and subsequently blocked with 1.5% normal goat serum in PBS for 1 h at RT as before. Sections were simultaneously incubated overnight at 4 °C in a humidified chamber with the rabbit polyclonal anti-IL-16 antibody (1:100) and a rat monoclonal anti-Ly6G antibody (1:200; Bio X Cell, BE0075-1). Negative-control sections were incubated under the same conditions in blocking solution without primary antibodies (supplementary file 1). The following day, sections were washed three times with PBS and incubated for 1 h at RT with Alexa Fluor 488-conjugated goat anti-rabbit IgG (1:250) and Alexa Fluor 546-conjugated goat anti-rat IgG (1:500). Sections were then washed three times with PBS, counterstained with 1 µg/mL DAPI for 10 min at RT, washed three additional times with PBS, and mounted using Fluoromount™ mounting medium.

Slides were observed in a Nikon Eclipse 80i epifluorescence microscope (Nikon Instruments Inc), equipped with a Nikon Intensilight-C-HGFI fluorescence lamp and with the appropriate fluorescence filters set for FITC, G2A or DAPI. Images were acquired with a Nikon DS-Ri1 digital camera. Negative controls were observed at the same exposure time used in the corresponding experiments. To obtained a more detailed view of tissue morphology, in transverse sections of sciatic nerves, bright-field images were overlaid with the corresponding IL-16 or CD4 immunofluorescence images. A Leica TCS SP8 X spectral confocal laser-scanning microscope equipped with a 405-nm diode laser and a white-light laser providing freely selectable excitation wavelengths between 470 and 670 nm was used. Images were acquired and processed using Leica Application Suite X software.

For quantification of DRG neurons labelled with the anti-IL-16 antibody, three animals per group (sham or injured) were analysed. From each sample, two images acquired at 100 × magnification were selected. Initially, neurons were considered as IL-16^+^ or IL-16^−^. In IL-16^+^ neurons, the smallest and largest diameters of each neuron were manually measured by using ImageJ/Fiji software and each neuron was classified by using the average of both measurements. Neurons were considered as small when this mean was < 22 µm, medium for a diameter between 22 and 34 µm or large for values greater than 34 µm [19].

In nerves, immunoreactive areas for IL-16 and CD4 obtained with the epifluorescence microscope were quantified by using ImageJ/Fiji software. Composite images containing either IL-16 or CD4 immunolabelling together with DAPI nuclear counterstaining acquired with 10× magnification were used. The green channel, corresponding to IL-16 or CD4 immunoreactivity, and the blue channel, corresponding to DAPI staining, were separated and converted into individual 8-bit grayscale images. For the green-channel images, intensity threshold was manually adjusted to include only pixels corresponding to visually identifiable immunoreactivity while minimizing the inclusion of background fluorescence. These images were subsequently converted into binary masks, and the total area occupied by the remaining positive pixels in the resulting binary mask was then quantified. To determine the total area of the sciatic nerve, the grayscale image corresponding to the DAPI channel was used as the reference. The distribution of DAPI-positive nuclei allowed to identify tissue boundaries to define the region analysed that was manually delineated by tracing the outer contour of the nerve based on the profile defined by the nuclear signal.

For IL-16, an additional analysis was performed in the proximal segment of the sciatic nerve located between the DRG and the most proximal ligature of the nerve. A segment of 1-mm long, close to the injury site was divided into 5 consecutive regions of 200 µm each was analysed following the procedure described above. Finally, IL-16 or CD4 immunoreactivity was expressed as the percentage of the analyzed nerve area occupied by the positive signal.

### Quantitative Real-Time Polymerase Chain Reaction (qRT-PCR)

Samples came from lumbar DRGs (L3-L6) ipsilateral to the ligated sciatic nerve as well as areas (7–9 mm approximately) of the sciatic nerve where the sham procedure or the ligatures had been done 2 days before. In ligated nerves, the knots were manually dissected off. After harvesting, tissues were immediately frozen in liquid nitrogen and stored at − 80 °C.

Total RNA was isolated by using EZNA Total RNA Kit I (Omega Bio-tek) and RNA concentration was determined using Nanodrop One (ThermoFisher Scientific). The first-strand cDNA was synthesized from 60 to 120 ng of total RNA priming by oligo(dT) using PrimeScript RT Reagent kit (Takara) and was kept at − 20 °C. Real-time quantitative PCR was performed with 30 ng of cDNA on a QuantStudio 5 thermal cycler (ThermoFisher Scientific) using SYBR Green and specific primers for the mouse gene of *Il16* forward 5′ GCC ATT CAG CCT ACA CCA GT 3′ and reverse 5′ GAC CAG AAA ATC GTC CTC CA 3’ [20], for the mouse gene of caspase-3 (*Casp3*) forward 5′ CTC GCT CTG GTA CGG ATG TG 3′ and reverse 5′ TCC CAT AAA TGA CCC CTT CAT CA 3′ [21], and for the β-actin (*Actb*) gene forward 5′ GCA GCT CCT TCG TTG CCG GT 3′ and reverse 5′ TAC AGC CCG GGG AGC ATC GT 3′. The cycling conditions consisted of an initial incubation at 50 °C for 2 min and denaturation at 95 °C for 10 min, followed by 40 cycles at 95 °C for 15 s and 60 °C for 1 min. Melting curve analysis was performed from 60 °C to 95 °C to confirm amplification specificity. *Actb* was used as housekeeping gene and data were analysed by the 2^−∆∆Ct^ method [22]. Each individual sample came from 4 DRGs or 1 sciatic nerve and the assay was performed in triplicate.

### Caspase-3 Activity

Lumbar DRGs (L3-L5) and sciatic nerve samples were harvested and kept as described in 2.7. DRG or sciatic nerves were homogenized in buffer (0.1 M Tris, 0.15 M NaCl, 0.5% CTAB, Fluka) with protease inhibitor (1 tablet/1.5 ml distilled water, Complete Mini Roche Diagnostics). In each case, 9 DRG or 4 sciatic nerves were used per sample. 3 μl of buffer per mg of tissue were added and tissues were homogenized with a Minicraft MB730 (Elmatik), centrifuged (15,000 g, 15 min, 4 °C) and their supernatant protein concentration measured by using a Nanodrop One (Thermo Fisher Scientific).

Caspase-3 activity was determined using Caspase-Glo 3/7 Assay (Promega, G8091), that measures the release of aminoluciferin, a substrate of luciferase, when the reaction occurs. 50 µl of the commercial assay mix, that provides the proluminescent caspase substrate, were added to 50 µl of samples containing 50 µg of protein. Luminescence was measured 1 h later by a spectrophotometer (Synergy H1, Biotek). Basal activity was calculated by adding the homogenization buffer, instead of tissue sample, and further subtracted from each reading. In parallel, as positive control, caspase-3 activity was also measured in purified enzyme caspase-3 (ENZO, ALX-201–059) samples.

### Statistical Analysis

Statistical analyses were performed using the GraphPad Prism software, version 6. Mean values and their corresponding standard errors were calculated. Data normally distributed, the Student’s t test was used to compare means of two groups whose data were followed a normal distribution, such as data coming from qRT-PCR, caspase-3 activity, % of IL-16^+^ or CD4^+^ surface in nerve. When more than two groups normally distributed were compared, an initial one-way ANOVA was performed when only one variable was considered, as occurred when characterizing IL-16^+^ DRG neurons in relation to their size. A two-way ANOVA was applied when two variables were considered such as hind paw ipsilateral or contralateral to the ligated sciatic nerve in behavioural studies performed in the unilateral hot plate test. These were followed by the Tukey’s post hoc test to establish differences. Data not normally distributed such as those obtained by the von Frey test were initially analysed by the Kruskall-Wallis test and differences further established by the Dunn’s test. Statistical significance was set at *p* < 0.05.

## Results

### Thermal Hyperalgesia and Mechanical Allodynia Evoked by Sciatic Ligation is Dose-Dependently Reversed after Neutralization of IL-16 or Its Receptor CD4

**Fig. 1:**
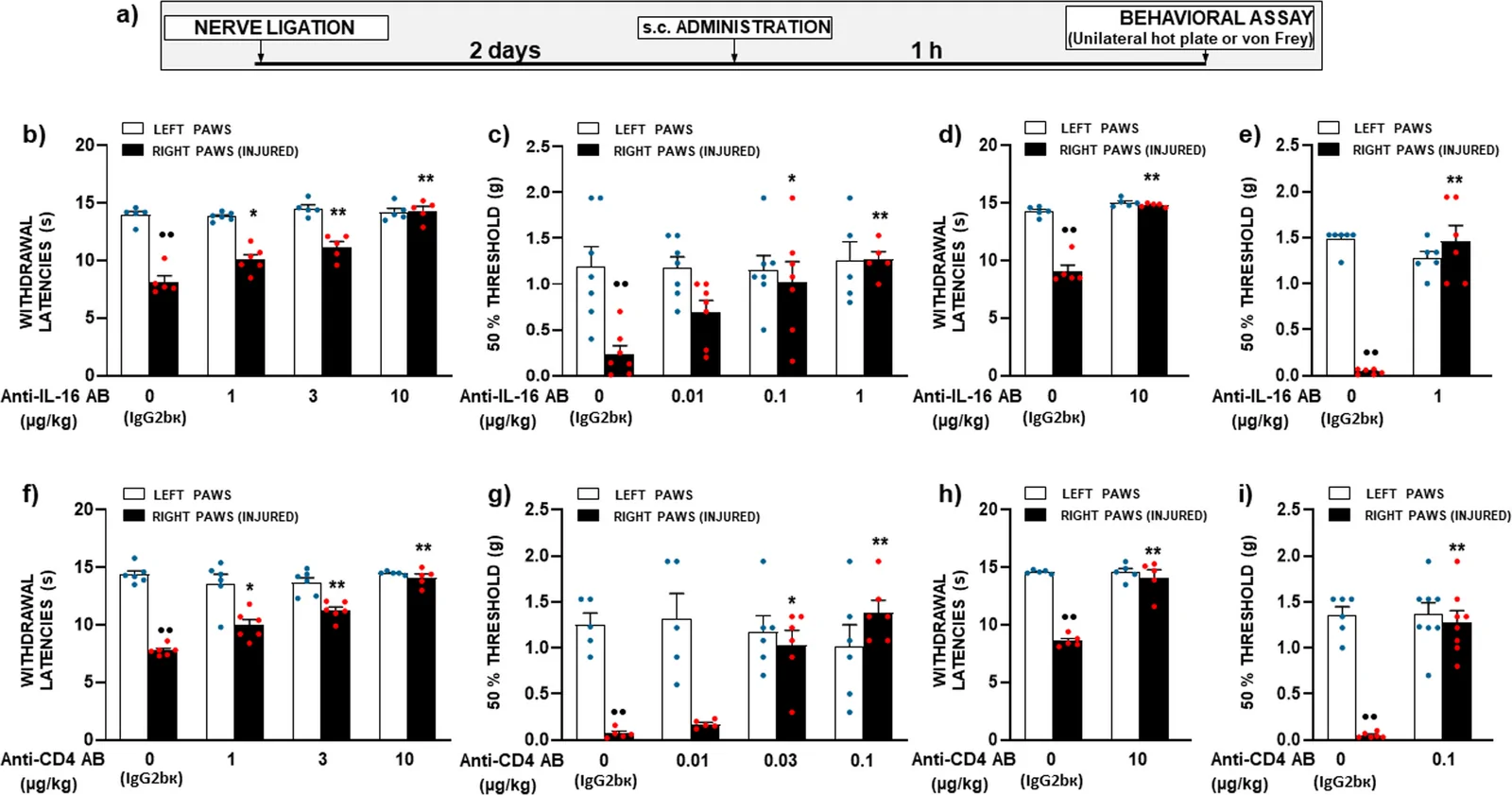
Effects of systemic treatment with an anti-IL-16 or an anti-CD4 antibody on the hypernociception measured 2 days after sciatic ligation in male and female mice. **a** General schema of the experimental behavioural procedure. **b**, **c** The s.c. administration of an anti-IL-16 antibody 1 h before testing dose-dependently inhibited neuropathic thermal hyperalgesia (**b**; 1–10 µg/kg; *n* = 5–6) and mechanical allodynia (**c**; 0.01–1 µg/kg; *n* = 5–7) in male mice. **d**, **e** Inhibitory effect evoked in female mice after the s.c. administration of the anti-IL-16 antibody of neuropathic thermal hyperalgesia (**d**; 10 µ/kg; *n* = 5) and mechanical allodynia (**e**; 1 µg/kg; *n* = 6) 1 h before testing. **f**, **g** The s.c. administration of an anti-CD4 antibody 1 h before testing dose-dependently inhibited neuropathic thermal hyperalgesia (**f**; 1–10 µg/kg; *n* = 5–7) and mechanical allodynia (**g**; 0.01–0.1 µg/kg; *n* = 5–6) in male mice. **h**, **i** Inhibitory effect evoked in female mice after the s.c. administration of the anti-CD4 antibody of neuropathic thermal hyperalgesia (**h**; 10 µ/kg; *n* = 5) and mechanical allodynia (**i**; 0.1 µg/kg; *n* = 6–8) 1 h before testing

The triple ligation of sciatic nerve evoked a rapid and consistent painful reaction in mice that was measured by thermal and mechanical behavioural methods. Thermal hyperalgesia was detected by the unilateral hot plate test 2 days after nerve injury, being withdrawal latencies reduced from values of about 14 s in contralateral paws and 8–9 s in the affected ones (Fig. 1b). This thermal hyperalgesia from neuropathic origin was dose-dependently reduced after the s.c. administration of an anti-IL-16 antibody (1–10 µg/kg) 1 h before testing (Fig. 1b). Nerve injury also induced clear-cut mechanical allodynia measured by the von Frey test, with this parameter also dose-dependently prevented after the s.c. injection of extremely low doses of this anti-IL-16 antibody (0.01–1 µg/kg; 1 h before testing) (Fig. 1c).

The involvement of CD4, the main receptor on which IL-16 exerts its effects, was assessed by administering an anti-CD4 antibody. Both thermal hyperalgesia (Fig. 1f) and mechanical allodynia (Fig. 1g) evoked by nerve ligation were dose-dependently inhibited after the administration of the anti-CD4 antibody. As occurred with the anti-IL-16 antibody, the doses required to avoid mechanical allodynia (0.01–0.1 µg/kg) were lower than those needed to inhibit thermal hyperalgesia (1–10 µg/kg).

In order to elucidate if these antihypernociceptive responses could be related to gender, experiments were also performed in female mice 2 days after nerve ligation. Initially, the degree of thermal hyperalgesia and mechanical allodynia measured in females was very similar to that observed in males. Furthermore, the s.c. administration of doses of anti-IL-16 antibody effective to completely prevent hyperalgesia and allodynia in male mice also counteracted these nociceptive parameters in female mice in the unilateral hot plate (Fig. 1d) or the von Frey test (Fig. 1e), respectively. A similar result was obtained when studying the effects of the anti-CD4 antibody either on thermal hyperalgesia (Fig. 1h) or mechanical allodynia (Fig. 1i).

**Fig. 2:**
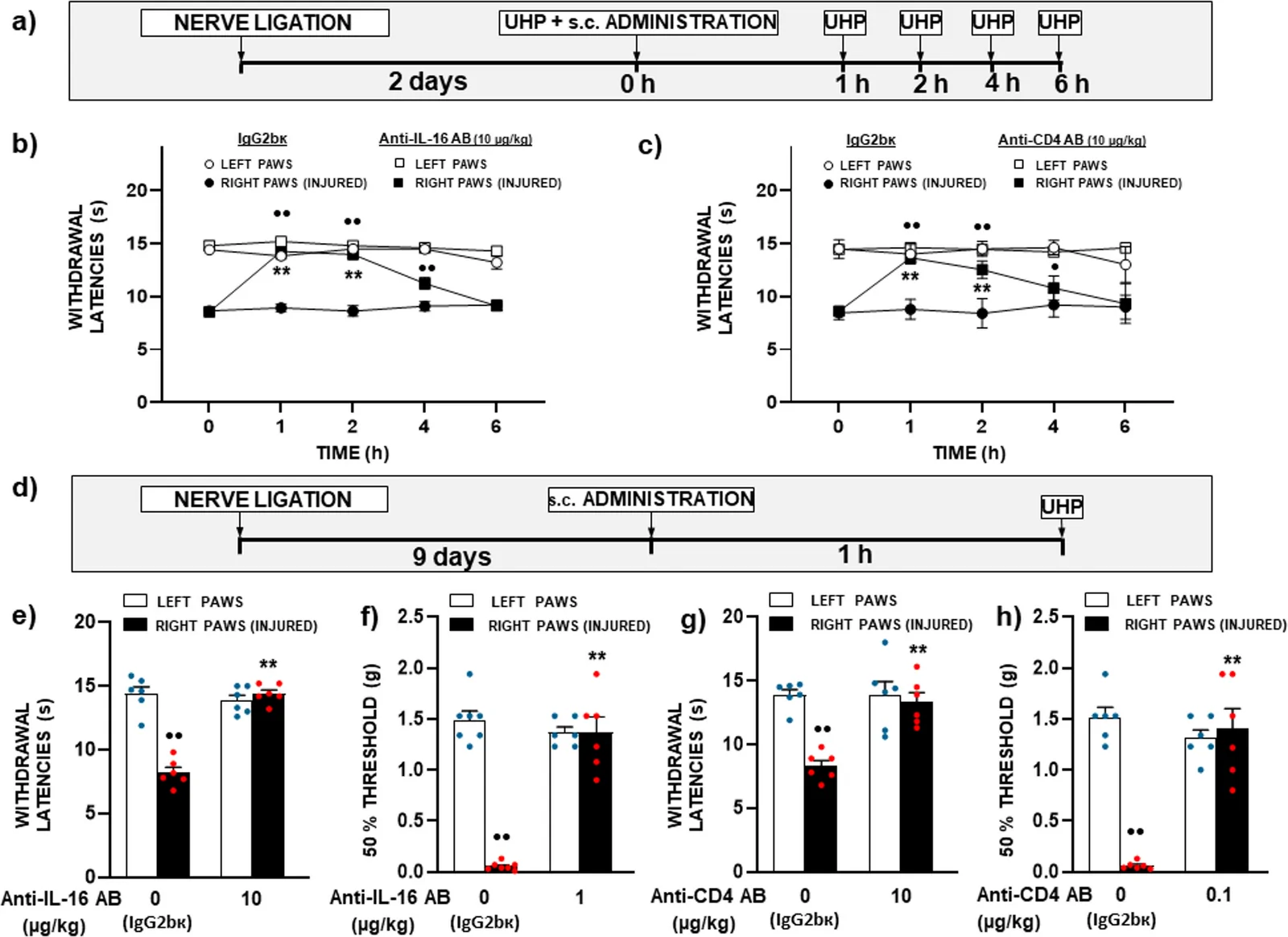
Time course of anti-IL-16 and anti-CD4 antibodies on thermal hyperalgesia measured 2 days after sciatic ligation in mice. Effects of systemic administration of an anti-IL-16 or an anti-CD4 antibody on hypernociception measured 9 days after sciatic ligation. **a** Schema of time course of the study of the antihyperalgesic effects induced by the anti-IL-16 or anti-CD4 antibodies in mice submitted to nerve ligation 2 days before. **b**, **c** Withdrawal latencies measured in mice 2 days after nerve ligation by the unilateral hot plate test 0, 1, 2, 4 and 6 h after the s.c. administration of anti-IL16 antibody (**b**; 10 µg/kg; *n* = 6), an anti-CD4 antibody (**c**; 10 µg/kg; *n* = 6) or the corresponding IgG2bk isotype (**b**,**c**; IgG2bk 10 µg/kg; *n* = 6 in each case). Each point represents the mean and corresponding standard error. • *p* < 0.05, •• *p* < 0.01 compared with the measure done at time 0, before drug administration; ** *p* < 0.01 compared with right paws of mice treated with IgG2bк isotype at the corresponding time, two-way ANOVA followed by Tukey’s test

The duration of the antinociceptive effects induced by both antibodies was assessed by the unilateral hot plate test. The antihyperalgesic effect evoked by the higher dose assayed of the anti-IL-16 antibody (10 µg/kg) was maximal 1 h after its s.c. administration, remained unaltered 1 h later, started to decrease 4 h after injection and became non-significant once 6 h had elapsed (Fig. 2b). The time course of the antihyperalgesic effect evoked by the anti-CD4 antibody was slightly shorter and already disappeared 4 h after administration (Fig. 2c).

The last behavioural approach performed to characterize the antihypernociceptive effects evoked by the blockade of anti-IL-16 and the anti-CD4 antibodies in our experimental model was to determine whether these effects could be exclusively related to the initial phases involved in the instauration of neuropathic pain measured at day 2 or if they could also come into play once hyperalgesia and allodynia are established at long-term. With this aim, we studied the effects of antihyperalgesic or antiallodynic doses of both antibodies in a group of male mice in which the ligation of nerves was performed 9 days before the assay. As shown in Fig. 2e–h, the hyperalgesic and allodynic effects measured at this time were of a similar degree than those observed 2 days after surgery. Furthermore, the maximal doses of the anti-IL-16 antibody used to inhibit thermal hyperalgesia (10 µg/kg; Fig. 2e) or mechanical allodynia (1 µg/kg; Fig. 2f) when tested 2 days after nerve injury, continued to completely revert both effects at day 9. A similar result was obtained when measuring the effect of the anti-CD4 antibody, since the effect of the administration of the maximal dose effective at day 2 also completely counteracted the hyperalgesic (10 µg/kg; Fig. 2g) and allodynic (0.1 µg/kg; Fig. 2h) effects measured 9 days after nerve injury.

### Peripheral Changes in the Expression of Il16 and Casp3 mRNA in Sciatic Nerve and DRGs in Response to Sciatic Ligation

**Fig. 3:**
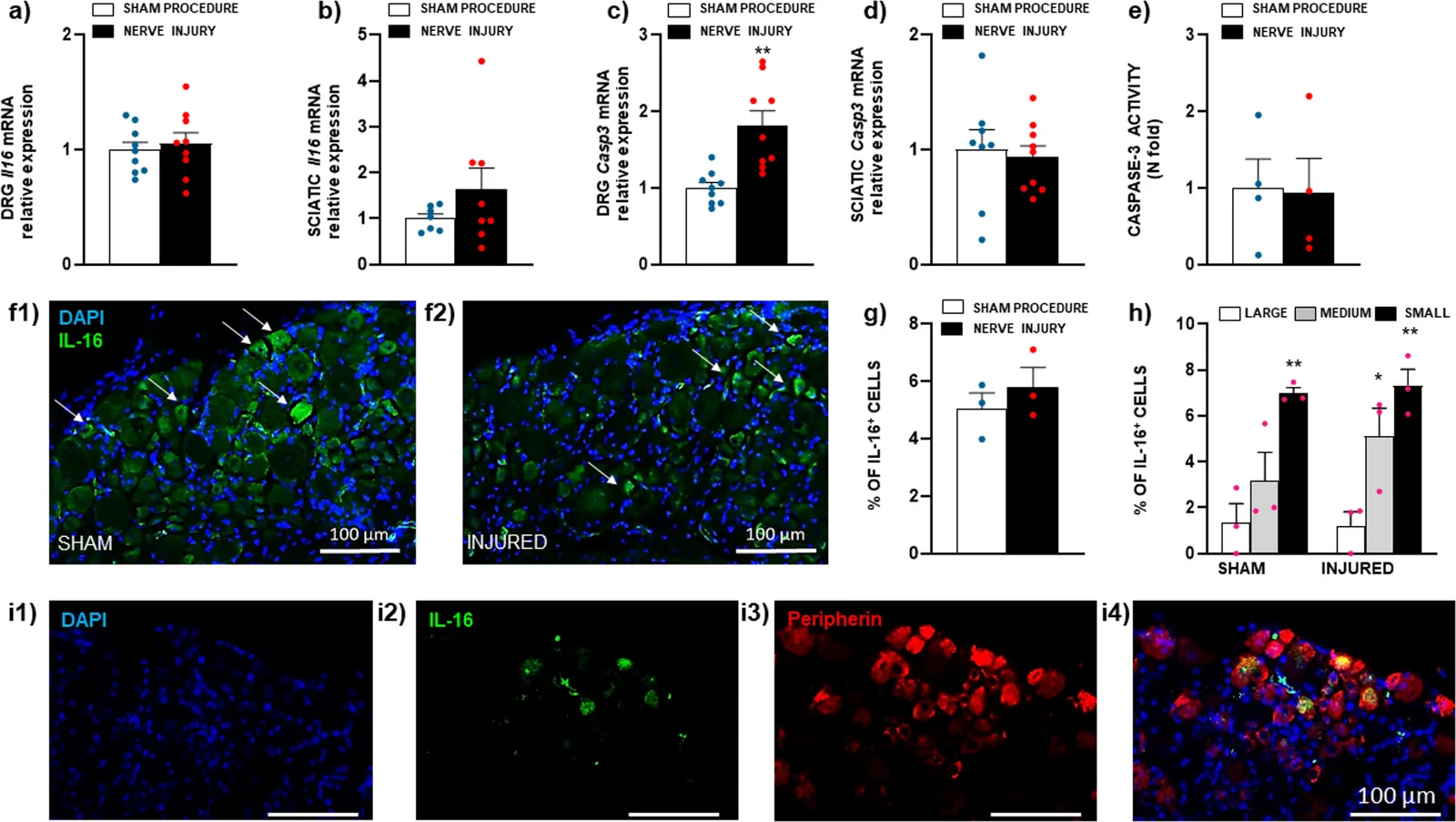
Effects on *Il16* and *Casp3* mRNA expression in DRGs and nerves two days after sciatic ligation. Measurement of caspase-3 activity and IL-16 immunofluorescence in DRGs of mice submitted to sham surgery or sciatic ligation. **a**–**b** *Il16* mRNA expression measured by qRT-PCR in DRGs (**a**; *n* = 9) or sciatic nerves (**b**; *n* = 7–8) coming from mice submitted to the sham surgical procedure or to sciatic nerve ligation. **c**–**d** *Casp3* mRNA expression measured by qRT-PCR in DRGs (**c**; *n* = 9) or sciatic nerves (**d**; *n* = 8–9) coming from mice submitted to the sham surgical procedure or to sciatic nerve ligation. **e** Caspase-3 activity measured in DRG homogenates coming from mice submitted to sham surgery or sciatic ligation 2 days before (*n* = 4). Means and their corresponding standard errors are represented. ** *p* < 0.01 compared with the value obtained in mice submitted to sham procedure, Student’s *t* test. **f** Representative example of IL-16 labelling by immunofluorescence in a DRG coming from a mouse submitted to sham surgery (f1) or to nerve ligation 2 days before (f2). Some IL-16^+^ neurons are indicated with arrows. **g** Percentage of IL-16^+^ cells quantified in DRG sections coming from mice submitted to sham surgery or sciatic ligation (*n* = 3). **h** Percentage of IL-16^+^ cells classified as large, medium or small in mice submitted to sham surgery (*n* = 3) or sciatic ligation (*n* = 3) 2 days before. In **g** and **h**, means and their corresponding standard errors are represented. * *p* < 0.05, ** *p* < 0.01 compared with the number of IL-16^+^ large DRG cells, one-way ANOVA followed by Tukey’s test. **i** Representative example of an image of DAPI (i1), IL-16 (i2) and peripherin (i3) labelling in DRG together with their merged image (i4) showing the colocalization of IL-16 and peripherin (yellow)

The assessment of possible changes in the expression of *Il16* mRNA at the peripheral nociceptive system in response to nerve injury was explored in qRT-PCR experiments performed in sciatic nerves or DRGs of mice submitted to sciatic ligation or sham procedure 2 days before. No change in the relative expression of this gene was found in these two sites (Fig. 3a–b). Alternatively, a putative involvement of IL-16 at nervous level could be, instead, related to a greater presence of caspase-3, the enzyme responsible for its activation. The influence of nerve constriction on the expression of *Casp3* mRNA was measured in nerves and DRGs and, as shown in Fig. 3c, a significant up-regulation was observed in DRGs of mice submitted to nerve injury compared to control mice, whereas its levels remained unaltered in sciatic nerves (Fig. 3d). Based on this result, we tried to detect a possible increased expression of IL-16 both in DRGs and in the injured portion of nerves.

### Enhanced Caspase-3 Activity and Increased Presence of IL-16 at Injured Nerves

Two experimental approaches were designed in order to explore whether the increased expression of *Casp3* mRNA in DRGs could lead to an enhanced presence of IL-16 in peripheral neurons. Initially, enzymatic caspase-3 activity was assessed in homogenates coming from DRGs and injured nerves. Results showed that the activity of caspase-3 remained unaltered in DRGs after nerve ligation (Fig. 3e). Immunofluorescence experiments to detect and measure the presence of IL-16 protein in L4 DRGs demonstrated the expression of IL-16 in a small and similar percentage of DRG neurons, about 5% of total neurons counted, either from control (Fig. 3f1, Fig. 3g) and injured mice (Fig. 3f2, Fig. 3g). Negative controls without primary antibody were observed and no fluorescence was detected (supplementary file 1). When classifying these IL-16^+^ cells in function of their sizes, we noticed the expression of this interleukin in small (diameter < 22 µm), medium (diameter 22–34 µm) and large (diameter > 34 µm) neurons*.* However, the frequency of IL-16 expression was different among these three categories, being the percentage of large neurons expressing IL-16 extremely low, about 1% of the total number of large cells counted both in mice submitted to sham surgery or nerve-injured mice (Fig. 3h). In contrast, the percentage of IL-16^+^ cells among medium and small DRG neurons was 7% in small cells and 3 to 5% in medium sized neurons in DRG from control or injured nerves, respectively (Fig. 3h). Thus, the number of small or medium neurons expressing IL-16 was about 10 times higher than in large ones, although its presence remained invariable after nerve injury. Confirming this view, double immunofluorescence assays showed that DRG neurons expressing IL-16 were also labelled by a peripherin antibody (Fig. 3i).

**Fig. 4:**
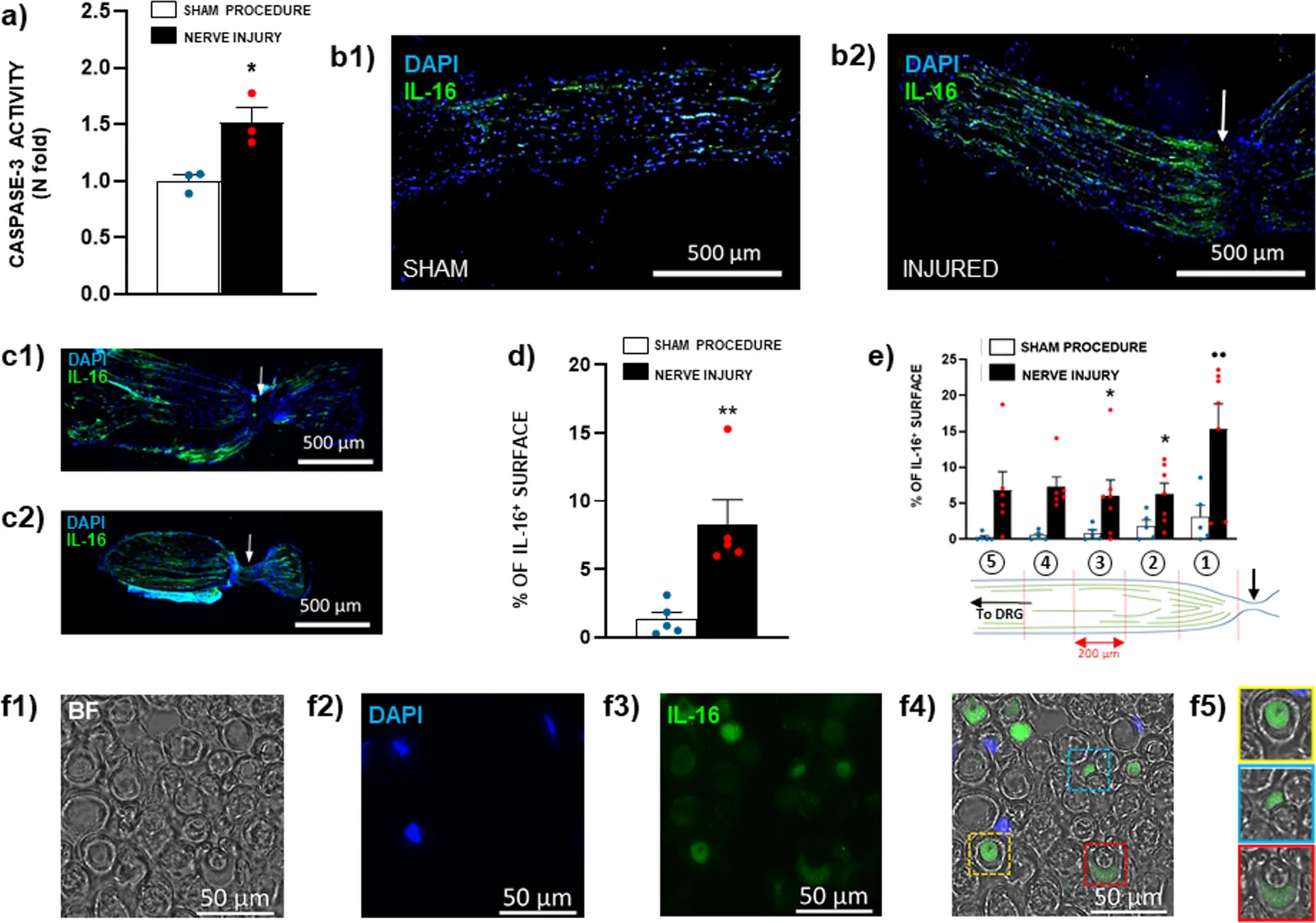
Measurement of caspase-3 activity and IL-16 immunofluorescence in sciatic nerves of mice submitted to sham surgery or sciatic ligation. **a** Caspase-3 activity measured in nerve homogenates coming from mice submitted to sham surgery or sciatic ligation 2 days before (*n* = 3). **b** Representative example of IL-16 labelling by immunofluorescence in a nerve coming from a mouse submitted to sham surgery (b1) or nerve ligation (b2) 2 days before. The image in b2 corresponds to the proximal side of the ligature closest to DRG. The white arrow indicates the ligation site. (**c**) Examples of IL-16 labelling in the second (c1) and third (c2) more distal ligatures. The white arrow indicates the ligation site. **d** Percentage of IL-16 immunoreactive surface measured in nerve sections coming from mice submitted to sham surgery or sciatic ligation (*n* = 5). **e** Bars of the upper graph represent the % of IL-16 immunoreactive surface measured in five 200-µm segments of sciatic nerves corresponding to the proximal area close to the first ligature (*n* = 5). In control mice submitted to sham surgery, an equivalent area of intact nerve was analysed (*n* = 5). Below, schematic draw showing the five sections in which the studied area was divided, being number 1 assigned to the segment closer to ligation. Green traits graphically represent IL-16^+^ axons. **f** IL-16 expression shown in a cross section performed at the first ligation of a sciatic nerve proximal to DRG. Bright field image without staining to visualize the histological structure with axon profiles and myelin sheaths (f1), DAPI staining (f2) and IL-16 labelling (f3) are shown. A merged image of f1, f2 and f3 showing a more precise localization of IL-16 immunofluorescence appears in f4. f5 shows details of f4, in which IL-16 labelling can observed in a myelinated axon (upper, yellow frame), a possible unmyelinated fibre (middle, blue frame) and a Schwann cell (lower, red frame). In (**a**), (**d**) and (**e**), means and their corresponding standard errors are represented. In (**a**) and (**d**) * *p* < 0.05; ** *p* < 0.01 compared with values obtained in mice submitted to sham surgery, Student’s *t* test. In (**e**) •• *p* < 0.01 compared with the corresponding segment in mice submitted to sham surgery; * *p* < 0.05 compared with segment number 1 of ligated nerves, two-way ANOVA followed by Tukey’s test

Contrasting with the result obtained in DRGs, caspase activity was significantly increased at the injured segments of the sciatic nerve (Fig. 4a). Besides, results obtained in immunofluorescence experiments performed at the injured portion of nerves showed that IL-16 presence was clearly augmented at this level. The presence of IL-16 was minimal in nerves of control mice, where only scarce positive fibres were found (Fig. 4b1). In contrast, IL-16 detection was markedly increased at nerves two days after injury. This up-regulation was observed in all three ligatures and it seemed more remarkable in the first one more proximal to DRG (Fig. 4b2), although it was also evident in the second (Fig. 4c1) and third ligation (Fig. 4c2). In all cases, IL-16 immunoreactivity in damaged nerves followed a longitudinal distribution, suggesting its expression along fibres (Fig. 4b–c). The quantification of the immunoreactive surface present in the proximal portion of the first ligation more proximal to the DRG showed about 15% of IL-16^+^ area, contrasting with the 1.5% found in a similar surface of non-injured nerves (Fig. 4d). Since it seemed that IL-16 up-regulation was more intense at nerve portions more proximal to each ligature, we measured the immunoreactive surface present in the first five 200-µm nerve segments proximal to DRG of the first ligature. Effectively, the mean of the first portion closest to the ligation showed 15% of IL-16^+^ surface, whereas the signal diminished in the subsequent portions up to about 5% (Fig. 4e), a mean that was maintained even in the more distal of these five portions.

**Fig. 5:**
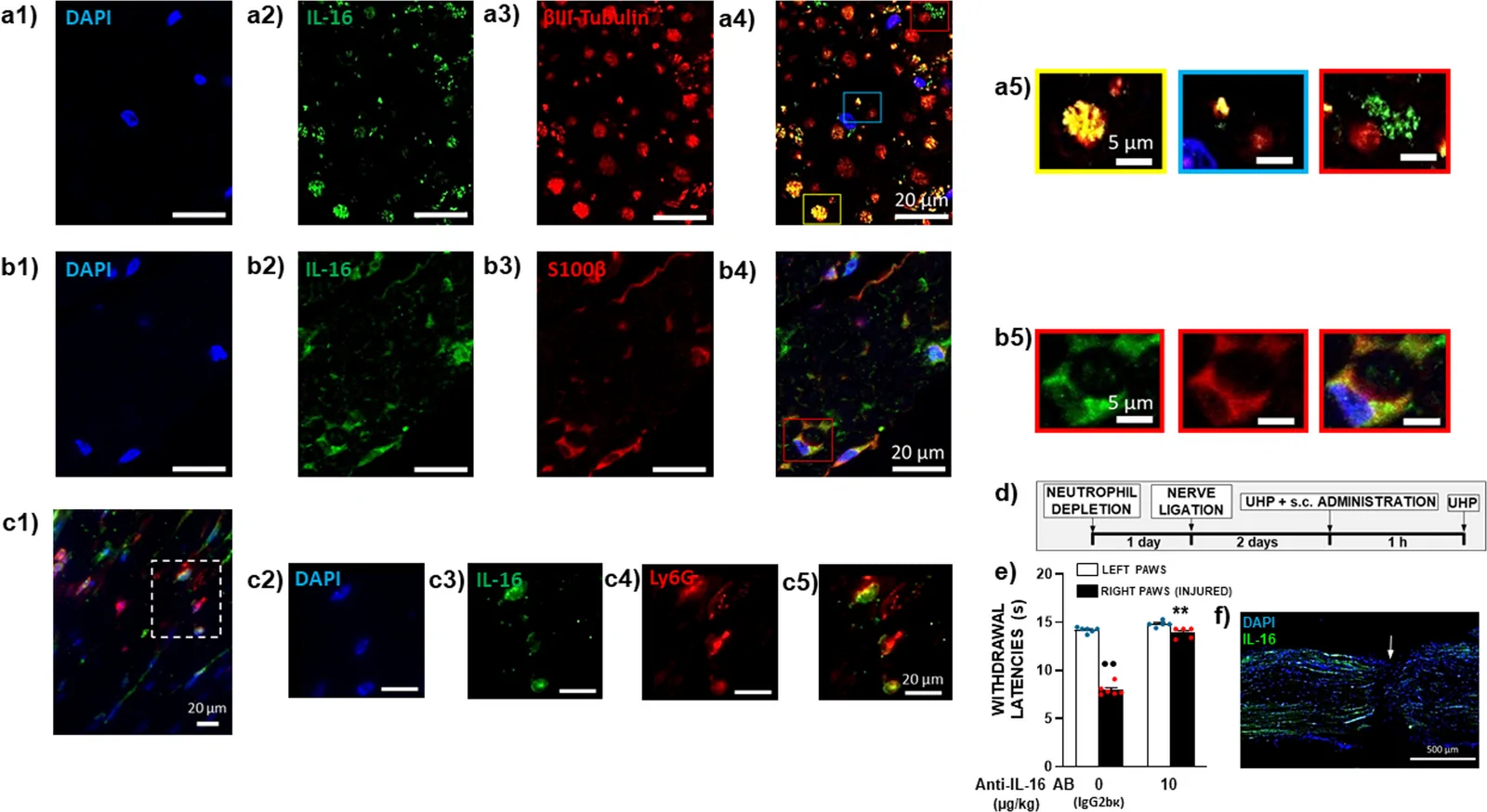
Expression of IL-16 in axons and Schwann cells of injured sciatic nerves. Lack of effect of neutrophil depletion on thermal hyperalgesia and IL-16 labelling in nerves. (**a1**–**a4**) Representative examples of confocal images of cross sections of injured sciatic nerves labelled with DAPI (blue, **a1**), IL-16 (green, **a2**) and the neural marker βIII-tubulin (red, **a3**). The merged image shows the colocalization of IL-16 and βIII-tubulin (yellow, **a4**), and higher-magnification images of the yellow, blue and red boxes are shown in **a5**). The yellow box shows an axon double-positive for IL-16 and βIII-tubulin (yellow). The blue box contains two small-diameter axons, one of them double-positive for IL-16 and βIII-tubulin (yellow) and the other only positive for βIII-tubulin (red). The red box indicates a βIII-tubulin-positive axon surrounded by IL-16 immunoreactivity with a shape compatible with a Schwann cell (green). (**b1**–**b4**) Confocal images corresponding to cross sections in injured sciatic nerves labelled with DAPI (blue, **b1**), IL-16 (green, **b2**) and the Schwann cell marker S100β (red, **b3**). The merged image shows the colocalization of IL-16 and S100β (yellow, **b4**). Higher-magnification images of the cell contained in the red box appear in **b5**, when labelled with IL-16 (green, left hand side), S100β (red, middle image) and the merged image with DAPI (yellow, right hand side). (**c1**) Representative merged image of IL-16, Ly6G and DAPI immunofluorescence in a longitudinal section coming from an injured nerve showing the presence of some neutrophils in a square remarked by a white dashed line. (**c2**–**c5**) Higher-magnification images of cells contained in the box, when labelled with DAPI (blue, **c2**), IL-16 (green, **c3**), Ly6G (red, **c4**) and the merged image showing the colocalization of IL-16 + and Ly6G + cells (yellow, **c5**). (**d**) Schematic representation of the experimental procedure used to evaluate the functional effect of an anti-IL-16 antibody in neutrophil-depleted mice submitted to sciatic nerve ligation 2 days before. (**e**) Decreased withdrawal latencies are shown in neutrophil-depleted mice 2 days after nerve ligation. The s.c. administration of the anti-IL-16 antibody (10 µg/kg) reversed this thermal hyperalgesia measured by the unilateral hot plate test 1 h later. Means and their corresponding standard errors are represented, •• *p* < 0.01 compared with the contralateral paw; ** *p* < 0.01 compared with values obtained in injured mice treated with IgG2bκ, two-way ANOVA followed by Tukey’s test. (**f**) Representative immunofluorescent merged image of IL-16^+^ traits (green) and DAPI nuclear staining (blue) in a longitudinal section of the ligated sciatic nerve from a neutrophil-depleted mouse

Trying to initially assess the localization of IL-16, immunofluorescence and bright field images were analysed in cross nerve sections of injured nerves. As shown in Fig. 4f, IL-16 immunoreactivity was mainly observed inside axons, some of which appeared surrounded by a visible myelin sheath (Fig. 4f4–f5) and some others were smaller and probably unmyelinated (Fig. 4f1-f5). Related to Schwann cells, some particular cases were also slightly labelled (Fig. 4f4–f5). Double immunofluorescence experiments were performed in transversal sections of ligated sciatic nerves in order to identify more accurately the type of nerve cells in which IL-16 expression occurred. As shown in Fig. 5, confocal microscopy photographs showed neural fibres with the expected red labelling due to βIII-tubulin antibody (Fig. 5a3) and some of them were also marked in green by the anti-IL-16 antibody (Fig. 5a2). The merge of both images together with the micrograph of nuclei stained in blue by DAPI (Fig. 5a4) showed yellow double labelling of some fibres (Fig. 5a5, left and central panels), thus demonstrating that IL-16 is expressed by a particular population of axons. In relation to the type of fibres expressing IL-16, it seems noteworthy that some of them show a diameter of about 1–2 µm, compatible with axons of C-fibres (Fig. 5a5, central panel) whereas other double labelled fibres were in the order of 5 µm, that corresponds to the size of Aδ fibres (Fig. 5a5, left panel). Finally, we also detected IL-16 labelling in structures close to βIII-tubulin^+^ fibres that could correspond to Schwann cells (Fig. 5a5, right panel). Trying to more directly check whether Schwann cells can, effectively, express IL-16, double immunofluorescence experiments were performed with the anti-IL-16 antibody and another one addressed against the glial marker S100β. As shown in Fig. 5b1-5, double labelling of both antibodies identified Schwann cells expressing IL-16.

Finally, since it was previously described that neutrophils are a type of immune cells that frequently express IL-16^+^ in response to inflammatory or infectious injuries [7, 11], we have specifically searched whether nerve injury could have elicited neutrophil infiltration, thus contributing to the presence of IL-16 after nerve injury. A double immunofluorescence assay with anti-Ly6G and anti-IL-16 antibodies was performed and a scarce population of neutrophils inside nerves was observed (Fig. 5c1), being some of them IL-16^+^ (Fig. 5c3–c5). Although the presence of IL-16^+^ neutrophils was, from a quantitative point of view, extremely low when compared with the expression of IL-16 in axons and Schwann cells, we designed some experiments addressed to explore whether this reduced proportion of IL-16 expressed by neutrophils could play a relevant functional role in this model of neuropathic pain. Initially, mice treated with a high dose of anti-Ly6G antibody previously reported to deplete neutrophils [13, 15] showed 2 days after nerve ligation a similar degree of thermal hyperalgesia than non-depleted mice, being this hypernociceptive reaction also completely prevented after the administration of the anti-IL-16 antibody (10 µg/kg; Fig. 5e). In addition, we observed that the pattern of IL-16 expression along axons and Schwann cells 2 day after ligation also appeared after neutrophil depletion (Fig. 5f). Thus, these results seem to indicate that neutrophils are not playing a relevant functional role in the peripheral hypernociceptive effect mediated by IL-16 2 days after nerve injury.

### Intra-sciatic IL-16 Evokes Hypernociception and its Local Neutralization at Injured Sciatic Nerve Prevents Neuropathic Pain

**Fig. 6:**
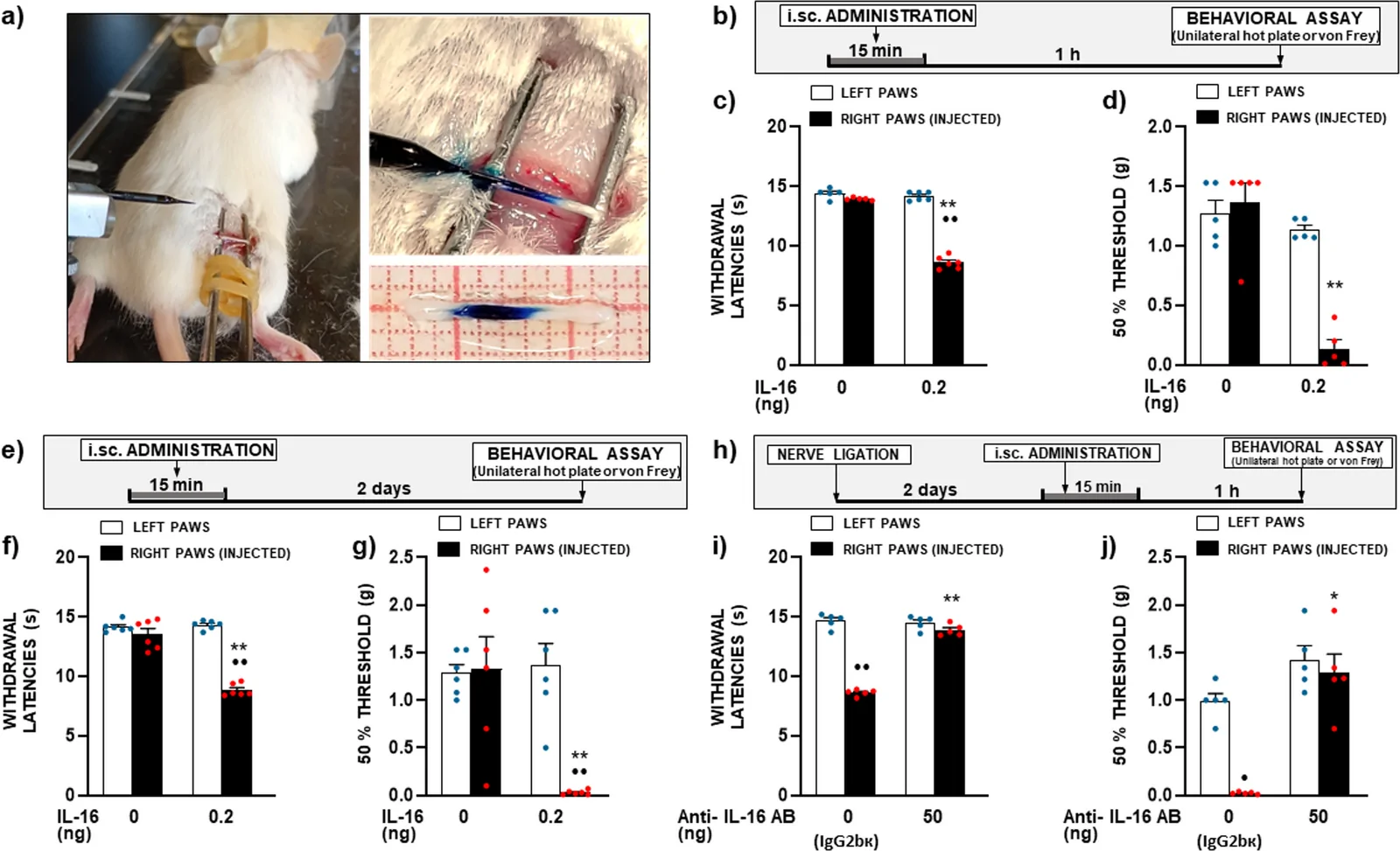
Hypernociceptive effects evoked by the intra-sciatic (i.sc.) administration of IL-16 and inhibition of hypernociception by i.sc. injection of an anti-IL-16 antibody in mice with nerve ligation. (**a**) Photographs of the intra-sciatic injection procedure performed with 1% methylene blue. On the left hand side, the sciatic nerve of the anesthetized mouse is hold with a surgical forceps and the micropipette loaded with the dye can be observed just before its introduction into the nerve. At the upper right hand side, a detailed view of the tip of the micropipette introduced into the nerve and the dye can be observed along a segment of the nerve. In the lower part of the right hand side, the removed nerve appears placed on a graph millimetre paper. (**b–d**) Effects of the i.sc. administration of 0.2 ng of IL-16 1 h after injection. A timeline schema is represented in (**b**), the effects of IL-16 or saline administration on thermal hyperalgesia appears in (**c**) (*n* = 5–6) and mechanical allodynia in (**d**) (*n* = 5). (**e**–**g**) Effects of the i.sc. administration of 0.2 ng of IL-16 2 days after injection. A timeline schema is represented in (**e**), the effects of IL-16 or saline administration on thermal hyperalgesia appears in (**f**) (*n* = 6) and mechanical allodynia in (**g**) (*n* = 6). (**h**–**j**) Behavioural testing of the effects induced after the intra-sciatic administration of the anti-IL-16 antibody in mice submitted to nerve ligation 2 days before. Timeline schema of the procedure (**h**). Prevention of thermal hyperalgesia (**i**; *n* = 5) and mechanical allodynia (**j**; *n* = 5) following the i.sc. administration of an anti-IL-16 antibody (50 ng; 1 h before testing) in mice submitted to nerve ligation 2 days before. Means and their corresponding standard errors are represented. •• *p* < 0.01 compared with their contralateral, non-injured, paws of mice; * *p* < 0.05, ** *p* < 0.01 compared with right paws of mice treated with saline or IgG2bk, two-way ANOVA followed by Tukey’s test (c,f,i) or Kruskall-Wallis followed by Dunn’s test (d,g,j)

Considering that our data pointed to a relevant role for IL-16 present into the injured portion of sciatic nerves, we measured the effects induced by the direct administration of IL-16 into the sciatic nerve (intra-sciatic administration, i.sc.). Although i.sc. administration was performed following a previously described protocol [16], since this is not a usual procedure of drug administration in our lab, we initially verified that the i.sc. injection of the dye methylene blue (0.1%) led to its exclusive presence inside the nerve. As shown in Fig. 6a, following the administration of 5 µl of solution at 20 µl/h for 15 min, the dye was only present into the nerve in a segment of approximately 4 mm long. Next, we checked whether the intra-sciatic injection of IL-16 could induce hypernociceptive responses 1 h after its administration and, also, whether they could also be measured 2 days after, the time chosen for the studies after nerve ligation. Initially, although this procedure must be performed under isoflurane anesthesia, control mice injected 1 h before with saline showed no motor or behavioural alterations and both thermal latencies and mechanical thresholds were undistinguishable from basal values obtained before treatment (Fig. 6c–d). The concentration of IL-16 used for intra-sciatic administration (0.04 ng/µl) was chosen based on the previous demonstration of its efficacy to evoke pronociceptive effects when administered peripherally into the paw of mice (1 ng/25 µl) [7] and was injected at a rate of 20 µl/h for a period of 15 min. Thus, the total amount of IL-16 injected into the nerve of each mouse during this period was a volume of 5 µl that represents a total dose of 0.2 ng. The assays performed by the unilateral hot plate and von Frey tests showed that this treatment produced clear-cut thermal hyperalgesia (Fig. 6c) and allodynia (Fig. 6d). In order to explore whether intraneural IL-16 could also be able to produce long-lasting hypernociception, the effect of this treatment was assessed in other group of mice 2 days after drug administration. The effects observed in behavioural tests indicated that one single intra-sciatic administration of IL-16 (0.2 ng) evokes both thermal hyperalgesia (Fig. 6f) and mechanical allodynia (Fig. 6g) 2 days after, whereas intra-sciatic administration of solvent did not modify thermal latencies or mechanical thresholds.

Next, we investigated the functional relevance of IL-16 expressed at the injured nerve by assaying the effect of the intra-sciatic administration of an anti-IL-16 antibody. As shown in figures, the administration of 50 ng (10 ng/µl at 20 µl/h for 15 min) of the anti-IL-16 antibody 1 h before testing completely abolished both thermal hyperalgesia (Fig. 6i) and mechanical allodynia (Fig. 6j) measured in mice 2 days after nerve ligation.

### CD4 Receptors Locally Expressed in the Sciatic Nerve are Involved in Hyperalgesia and Allodynia Evoked by Sciatic Ligation

**Fig. 7:**
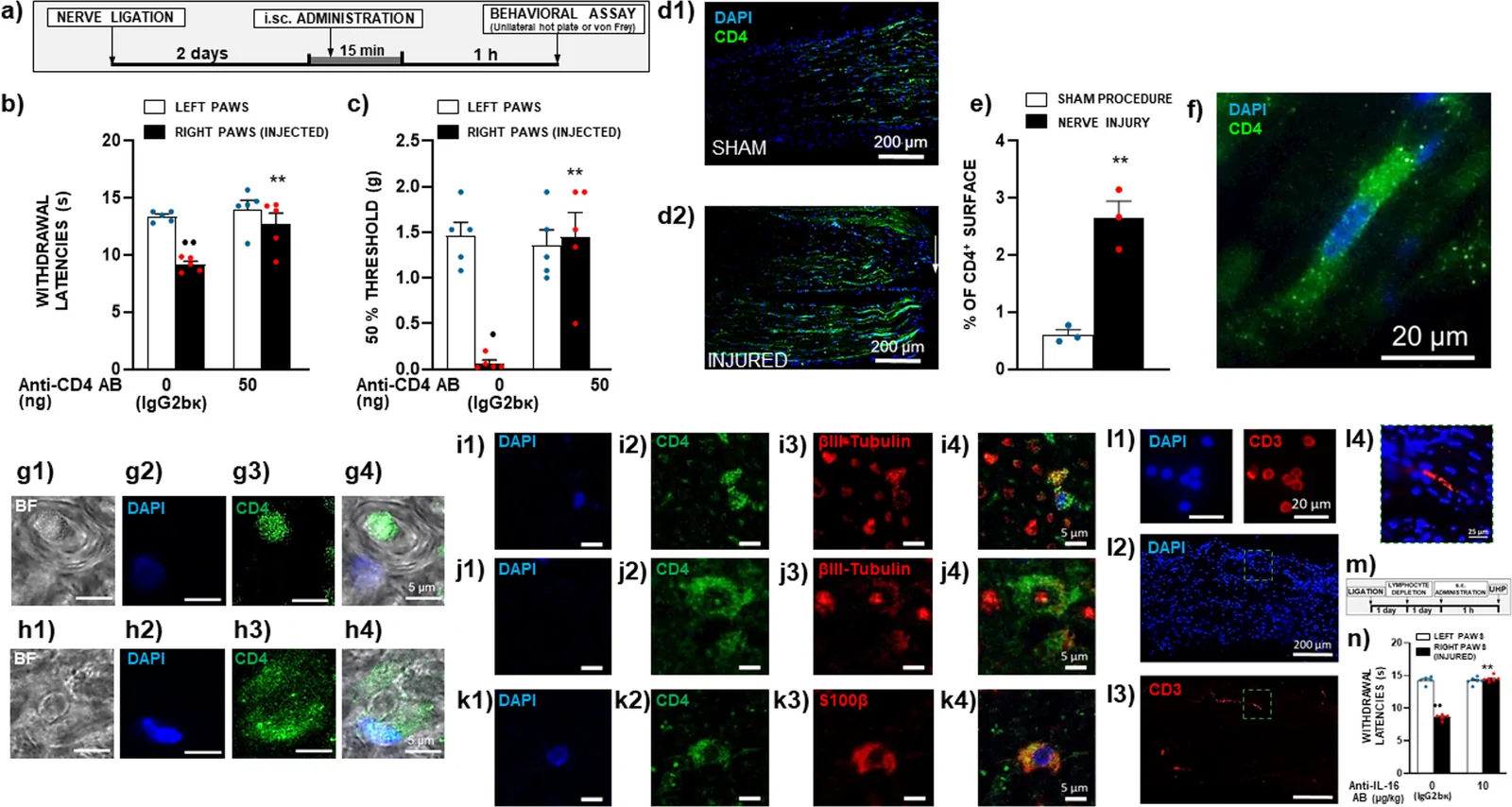
Inhibition of neuropathic hypernociception by i.sc. injection of an anti-CD4 antibody. Expression of CD4 receptors in sciatic nerves and up-regulation after nerve injury. Absence of CD3 labelling in the sciatic injured nerve and lack of effect of lymphocyte depletion on neuropathic thermal hyperalgesia. (**a**–**c**) Behavioural testing of the effects induced after the intra-sciatic administration of the anti-CD4 antibody in mice submitted to nerve ligation 2 days before. A timeline schema of the procedure is represented in (**a**). The i.sc. administration of an anti-CD4 antibody (50 ng; 1 h before testing) prevents thermal hyperalgesia (**b**; *n* = 5) and mechanical allodynia (**c**; *n* = 5) in mice submitted to nerve ligation 2 days before. In (**b** and **c**), means and their corresponding standard errors are represented, • *p* < 0.05, •• *p* < 0.01 compared with mice submitted to sham surgery; ** *p* < 0.01 compared with values obtained in injured mice treated with IgG2bκ, two-way ANOVA followed by Tukey’s test. (**d**) Representative example of CD4 labelling by immunofluorescence in a nerve coming from a mouse submitted to sham surgery (**d1**) or nerve ligation (**d2**) 2 days before. The image in **d2**) corresponds to the proximal side of the ligature closest to DRG. The white arrow indicates the ligation site. (**e**) Percentage of CD4 + immunoreactive surface measured in nerve sections coming from mice submitted to sham surgery or sciatic ligation (*n* = 3). Means and their corresponding standard errors are represented, ** *p* < 0.01 compared with mice submitted to sham surgery, Student’s *t* test. (**f**) Immunofluorescence image showing a CD4 + cell located in the sciatic nerve compatible with a Schwann cell with its nucleus stained with DAPI, (**g1**–**g4**) CD4 expression in a cross section performed at the first ligation of a sciatic nerve proximal to DRG showing a myelinated axon. Bright field image showing the histological structure compatible with the axon profile and its myelin sheath (**g1**), DAPI staining (blue, **g2**) and CD4 labelling (green, **g3**) are shown. The merged image (**g4**) shows a more precise localization of CD4 immunofluorescence. (**h1**–**h4**) CD4 expression in a cross section performed at the first ligation of a sciatic nerve proximal to DRG showing CD4 labelling in a Schwann cell surrounding a myelinated axon. Bright field image without staining shows the histological structure compatible with a Schwann cell surrounding an axon (**h1**), DAPI staining (blue, **h2**) and CD4 labelling (green, **h3**) are shown. The merged image (**h4**) shows a more precise localization of CD4 immunofluorescence. (**i**) Representative examples of confocal images of cross sections of injured sciatic nerves labelled with DAPI (blue, **i1**), CD4 (green, **i2**) and the neural marker βIII-tubulin (red, **i3**). The merged image shows the colocalization of CD4 and βIII-tubulin in an axon (**i4**, yellow). (**j**) Representative examples of confocal images of injured sciatic nerves labelled with DAPI (blue, **j1**), CD4 (green, **j2**) and the neural marker βIII-tubulin (red, **j3**). The merged image (**j4**) shows the labelling of an axon (red) surrounded by a CD4^+^ cell compatible with the shape of a Schwann cell (green). (**k**) Representative examples of confocal images of injured sciatic nerves labelled with DAPI (blue, **k1**), CD4 (green, **k2**), the Schwann cell marker S100β (red, **k3**). The merged image shows the colocalization of CD4 and S100β + Schwann cell (**k4**, yellow). (**l1**-**l4**) Immunofluorescence images of DAPI (blue) and CD3 (red) labelling in thymus cells (**l1**) and longitudinal nerve sections coming from mice submitted to nerve ligation 2 days before (**l2**-**l4**). Contrasting with the clear-cut lymphocyte morphology observed in the thymus (**l1**), no CD3 labelling is observed in nerve (**l2**-**l4**) and the sporadic red labelling signal that appeared in some nerve sections was not associated to DAPI-stained nuclei (Fig. 7**l4**). (**m**-**n**) Lack of effect of lymphocyte depletion on the hyperalgesic effect evoked by nerve ligation and on the antihyperalgesic effect evoked by the administration of an anti-IL-16 antibody. **m**) Schematic representation of the experimental procedure used to evaluate the functional effect of an anti-IL-16 antibody in lymphocyte-depleted mice submitted to sciatic nerve ligation. **n**) Decreased withdrawal latencies are shown in lymphocyte-depleted mice 2 days after nerve ligation. The s.c. administration of the anti-IL-16 antibody (10 µg/kg) reversed this thermal hyperalgesia measured by the unilateral hot plate test 1 h later. Means and their corresponding standard errors are represented, •• *p*<0.01 compared with the contralateral paw; ** *p*<0.01 compared with values obtained in injured mice treated with IgG2bκ, two-way ANOVA followed by Tukey’s test.

Considering that hyperalgesia and allodynia produced by nerve ligation was counteracted by systemic administration of an anti-CD4 antibody and that IL-16 neutralization at the injured level was able to inhibit neuropathic hypernociception, we focussed on the characterization of the role played by CD4 receptors expressed at the injured nerve. The acute intra-sciatic administration 1 h before testing of a single dose of anti-CD4 antibody (50 ng; injection of a solution of 10 ng/µl at 20 µl/h for 15 min) completely prevented neuropathic hyperalgesia (Fig. 7b) and allodynia (Fig. 7c) measured 2 days after nerve ligation, thus supporting the involvement of local CD4 receptors in both effects.

We next tried to identify and localize the presence of these receptors at the sciatic nerve by using immunofluorescence assays. In non-injured nerves, a not very abundant but specific CD4 labelling was detected (Fig. 7d1), while the observation of negative controls without primary antibody did not show fluorescence (Supplementary file 1). In contrast, a remarkable increase of CD4^+^ immunolabelling was found in ligated nerves (Fig. 7d2), and the quantification of CD4^+^ immunoreactive area demonstrated an important increase in injured nerves 2 days after ligation (Fig. 7e). Both in damaged and non-damaged nerves, the distribution of CD4 appeared following two patterns. Although a first analysis revealed the evident presence of longitudinal traits compatible with its expression in axons, shorter and localized immunoreactive structures with DAPI-stained nuclei were also detected, suggesting that CD4 expression can also occur in Schwann cells (Fig. 7f). In fact, the study of CD4 labelling in nerve cross sections of an injured nerve, confirmed its presence in axons surrounded by myelin sheaths and their corresponding Schwann cells (Fig. 7g1–g4). In addition, CD4 immunoreactivity was again detected in structures with typical Schwann cells morphology, located surrounding axons, with irregular shapes and their nuclei stained with DAPI (Fig. 7h1–h4).

Following a similar experimental strategy than that used to elucidate which type of nerve cells were labelled with IL-16, double immunofluorescence assays were performed in transversal sections of ligated sciatic nerves to determine the type of cells expressing CD4 in response to nerve injury. Images obtained by confocal microscopy showed that particular axons identified with an anti-βIII-tubulin antibody were also labelled with the anti-CD4 antibody (Fig. 7i1–i4). A detailed analysis revealed that some βIII-tubulin^+^ axons were surrounded by a CD4^+^ cell with a shape and localization compatible with Schwann cells (Fig. 7j1–j4). The performance of double immunofluorescence assays with an anti-S100β antibody showed that, effectively, some Schwann cells labelled with this glial marker were also expressing CD4 receptors (Fig. 7k1–k4). These results confirmed that both neurons and glial cells can express CD4 receptors in injured nerves.

Finally, although double immunofluorescence assays showed CD4 expression in neural structures, considering that the main type of body cells that express CD4 receptors are CD4^+^ lymphocytes and that the theoretical possibility of lymphocyte infiltration in injured nerves could exist, we assessed the presence of lymphocytes in damaged nerves. With this aim, we performed immunofluorescence assays with an anti-CD3 antibody in cross sections from injured nerves. Using the same experimental conditions in which thymus lymphocytes were clearly identified (Fig. 7l1), no CD3^+^ lymphocytes appeared in damaged nerves (Fig. 7l2-4). In fact, the scarce red labelling detected in nerves was never associated with DAPI-stained-nuclei, thus discarding that they could correspond to immune cells but rather to an occasional unspecific signal (Fig. 7l4). Furthermore, in order to assess the possible relevance of lymphocytes, a behavioural experiment was performed in mice after lymphocyte depletion. In this assay, a dose of anti-CD3 antibody (1 µg) previously used in our lab [13] and others [14] to evoke the reduction of circulating lymphocytes was administered. As shown in Fig. 7m–n, thermal hyperalgesia measured in response to nerve ligation remained unaltered after lymphocyte depletion and it was completely reverted after the administration of 10 µg/kg of the anti-IL-16 antibody (Fig. 7n). Thus, our results indicate that the presence of CD4 receptors in nerves cannot be attributed to a possible lymphocyte infiltration.

## Discussion

In the present experiments, we focus on the role played by peripheral IL-16 in a murine model of nerve damage and we show that local mechanisms related to this interleukin and CD4 receptors at the injured nerve can be involved in hypernociceptive responses. Initially, our data show that both thermal hyperalgesia and mechanical allodynia measured 2 days after sciatic ligation is prevented by the neutralization of IL-16 or CD4 receptors with the corresponding antibodies systemically administered, thus confirming the role played by IL-16 and CD4 receptors in neuropathic pain suggested by previous studies [8, 9].

Although the majority of experiments included in the present manuscript have been performed in male mice, since a previous report related to the involvement of IL-16 in a murine model of nerve transection 14 days before testing showed gender-related differences in the expression of spinal *Il16* mRNa [9], we have performed some key experiments in females to elucidate whether gender differences could modify the efficacy of the antibodies against IL-16 or CD4. Our data show that similar antinociceptive effects are produced in male and female mice in response to the administration of both antibodies, thus suggesting that gender may not be a key variable for the functional involvement of the IL-16/CD4 axis in neuropathic pain.

It has been previously described that different mechanisms might be involved in the establishment and the maintenance of this type of pain [23, 24]. Since the present study was carried out 2 days after nerve injury, we tried to elucidate whether the participation of IL-16 and CD4 receptors is limited to the early phase of instauration of neuropathic pain or could also play a role in the further maintenance mechanisms. With this aim, we assessed the efficacy of the neutralization of IL-16 or CD4 receptors in experiments performed 9 days after ligation. The obtaining of similar results 2 and 9 days after injury suggests that IL-16 and CD4 receptors are not only involved in the establishment of neuropathic painful processes but also in its maintenance.

In order to evaluate whether the participation of IL-16 in neuropathic nociception could be related to changes in IL-16 expression at peripheral level, qRT-PCR assays were performed in DRG and sciatic nerve to measure mRNA expression of *Il16* and *Casp3*, the main enzyme responsible of its synthesis [25]. Whereas *Il16* mRNA expression remained unmodified both in DRGs and nerves after ligation, *Casp3* mRNA was up-regulated at DRG level, but not in the injured portion of sciatic nerves. A recent study performed in rats described the augmentation of both *Il16* mRNA measured by PCR and IL-16 protein assessed by western blot in DRGs and nerves of rats 14 days after sciatic nerve ligation [8]. The discrepancy with our results could be mainly due to methodological differences, such as the species used (rat *vs* mouse) or the time after injury studied (14 days *vs* 2 days after injury). In any case, the increase in *Casp3* mRNA expression in DRG is in accordance with similar results obtained in different models of experimental nerve injury in rats. As such, increased values of *Casp3* mRNA has been described in DRG after L5/L6 spinal nerve ligation [26], spinal nerve transection [26, 27] or chronic constriction injury [28]. The increased expression of *Casp3* mRNA in DRG neurons was not accompanied by greater enzymatic activity of caspase-3 in DRG or an enhanced expression of IL-16 in DRG of mice with ligated nerves, thus suggesting that, in our experimental conditions, its expression at neuronal somas is not a crucial variable in response to peripheral nerve injury. In immunofluorescence studies, IL-16 expression was detected in about 5–6% DRG neurons, being the great majority of IL-16^+^ neurons small or medium sized. A double immunofluorescence assay performed by using the anti-IL-16 antibody together with an antibody against peripherin, a type III intermediate filament protein that specifically labels small sized neurons [29], confirmed that small and medium sized DRG neurons were the main types of DRG cells expressing IL-16. This presence of IL-16 in small and medium neurons seems compatible with a putative relationship between IL-16 and nociceptive transmission.

In contrast with the lack of effect of nerve ligation on IL-16 expression in DRGs, the presence of this cytokine was importantly augmented in the injured portion of nerves. Thus, IL-16 expression was dramatically augmented two days after nerve ligation, following a longitudinal pattern along the nerve, being this result in accordance with the up-regulation of IL-16 described in rat sciatic nerves 14 days after nerve ligation [8]. A more detailed analysis of bright field images performed in cross sections indicates that IL-16 is present inside axons, some of which were clearly myelinated. Also, a slight expression of IL-16 in Schwann cells located proximally to the injury was observed. These localizations were confirmed by performing double immunofluorescence experiments performed with βIII-tubulin or S100β, specific markers of axons [30] and Schwann cells [31], respectively. Apart from this expression in fibres and glial cells, the infiltration of IL-16^+^ neutrophils could also contribute to the increased presence of this cytokine, as occurs at the periphery in inflammatory [7] or infectious [11] settings. However, the global contribution of neutrophils to IL-16 nerve expression is not functionally relevant, since their depletion does not alter thermal hyperalgesia in injured mice.

In order to check whether the increased levels of IL-16 in the injured nerve may importantly contribute to neuropathic pain, it seemed necessary to demonstrate that the sole presence of IL-16 in a nerve may lead to hypernociceptive responses. The clear-cut hypernociceptive effect following the direct injection of this interleukin into the sciatic nerve strongly supported the possibility that IL-16 up-regulation in nerves could lead to pain sensitization. Furthermore, the intra-sciatic administration of a small dose of the anti-IL-16 antibody completely prevented allodynia and hyperalgesia measured in mice with constricted nerves, thus strongly suggesting that the local presence of IL-16 in nerve plays a relevant role in neuropathic nociception.

Once established the role played by IL-16 at the injured nerve, our next challenge was to detect the expression of the receptors activated by this cytokine in nerves. Certainly, there is not an extensive bibliographic support for the expression of CD4 receptors either in neurons or glial cells at peripheral nerves. In fact, a recent report even proposed the opposite idea arguing that HIV infection does not lead to viral destruction of nerve cells because neither DRG neurons nor Schwann cells express CD4 receptors [32]. Furthermore, although the detection of CD4 receptors has been reported in nerves after crush injury, their expression was associated with infiltrating macrophages and not nerve cells [33]. Probably, the experimental result that gives the strongest support to the functional presence of CD4 receptors in neurons is the description that a small proportion of DRG neurons responds with a rapid rise of intracellular calcium after their exposition to IL-16 [20]. In any case, the early hypernociceptive effect obtained in the present experiments 1 h after intra-sciatic administration of IL-16 observed in the present experiments prompted us to consider the possible participation of constitutive CD4 receptors expressed in the sciatic nerve. Initially, in order to confirm that local CD4 receptors were the target activated by IL-16 to evoke neuropathic pain, we administered an anti-CD4 antibody by intra-sciatic injection and we observed that its local presence in the nerve completely counteracts both hyperalgesia and allodynia produced in this setting. Besides the rapid behavioural response obtained after intra-sciatic injection of the anti-CD4 antibody, immunofluorescence experiments show their up-regulation in response to nerve injury. Micrographs of transversal sections of injured nerves suggested that expression of CD4 receptors could occur both in axons and Schwann cells, a fact that was confirmed by performing double labelling with anti-βIII-tubulin and anti-S100β antibodies. This CD4 receptor expression seems unrelated to the possible infiltration of lymphocytes, since, at least at this time, immunofluorescence assays showed that these immune cells were not present at injured nerves. In addition, both thermal hyperalgesia induced by nerve ligation and the analgesic effect evoked by the anti-IL-16 antibody remained unaltered in mice treated with a dose of an anti-CD3 antibody able to deplete circulating lymphocytes [13, 14]. Thus, our results indicate that the increased presence of CD4 receptors observed after nerve injury does not correspond to lymphocyte infiltration but to an up-regulation of CD4 receptors in peripheral nerves. These data point towards the same direction as previous reports indicating that the presence of CD4 receptors in the spinal cord of mice is increased in response to inflammatory [9] or neuropathic [10] injury. In this sense, our results indicate that the increase in the expression of CD4 receptors does not only occur at spinal level but also at peripheral nerves.

Overall, these behavioural results support the efficacy of the blockade of the IL-16/CD4 axis to prevent painful neuropathic symptoms in mice. Immunofluorescence assays showed the constitutive expression of IL-16 in a subpopulation of small and medium sized DRG neurons, the presence of CD4 receptors in a population of axons and Schwann cells of intact nerves and their up-regulation after nerve injury. In addition, results obtained after intra-sciatic administration of IL-16 further demonstrated the prominent modulatory properties of this cytokine on nociceptor excitability. Altogether, these data reinforce and complete previous observations related to the participation of IL-16 and CD4 receptors in pain and, particularly, in experimental neuropathic settings [8, 9]. In relation to a putative clinical translation, some reports have demonstrated increased IL-16 levels in serum of patients with diseases accompanied by painful symptoms, such as endometriosis [34], pancreatitis [35, 36], arthroplasty [37] or post-pandemic COVID-related chronic pain [38]. Focussing on neuropathic pain, only a recent manuscript describes that patients affected by trigeminal neuralgia show increased levels of IL-16 in cerebrospinal fluid [39]. Thus, our results could be interpreted as an initial laboratory effort to define innovative mechanisms addressed to manage pain responses. Tentatively, the wide clinical experience acquired during the past decades with the use of monoclonal antibodies could make conceivable a potential use of anti-IL-16 or anti-CD4 antibodies as new tools to counteract neuropathic pain symptoms. However, further clinical studies seem necessary to confirm or discard this possibility.

## Supplementary Information

Below is the link to the electronic supplementary material.

ESM 1 (PDF 390 KB)

## Data Availability

The datasets generated during the current study are available from the corresponding author on reasonable request.
