# Supplementary material for: Neural Expression of IL-16 Mediates Neuropathic Pain after Sciatic Injury in Mice
Source: Mol Neurobiol. 2026 Oct 22;63(1):987. doi: 10.1007/s12035-026-06280-2 (PMC13690839; doi:10.1007/s12035-026-06280-2)
Supplement: Supplementary file 1 [file 12035_2026_6280_MOESM1_ESM.pdf]

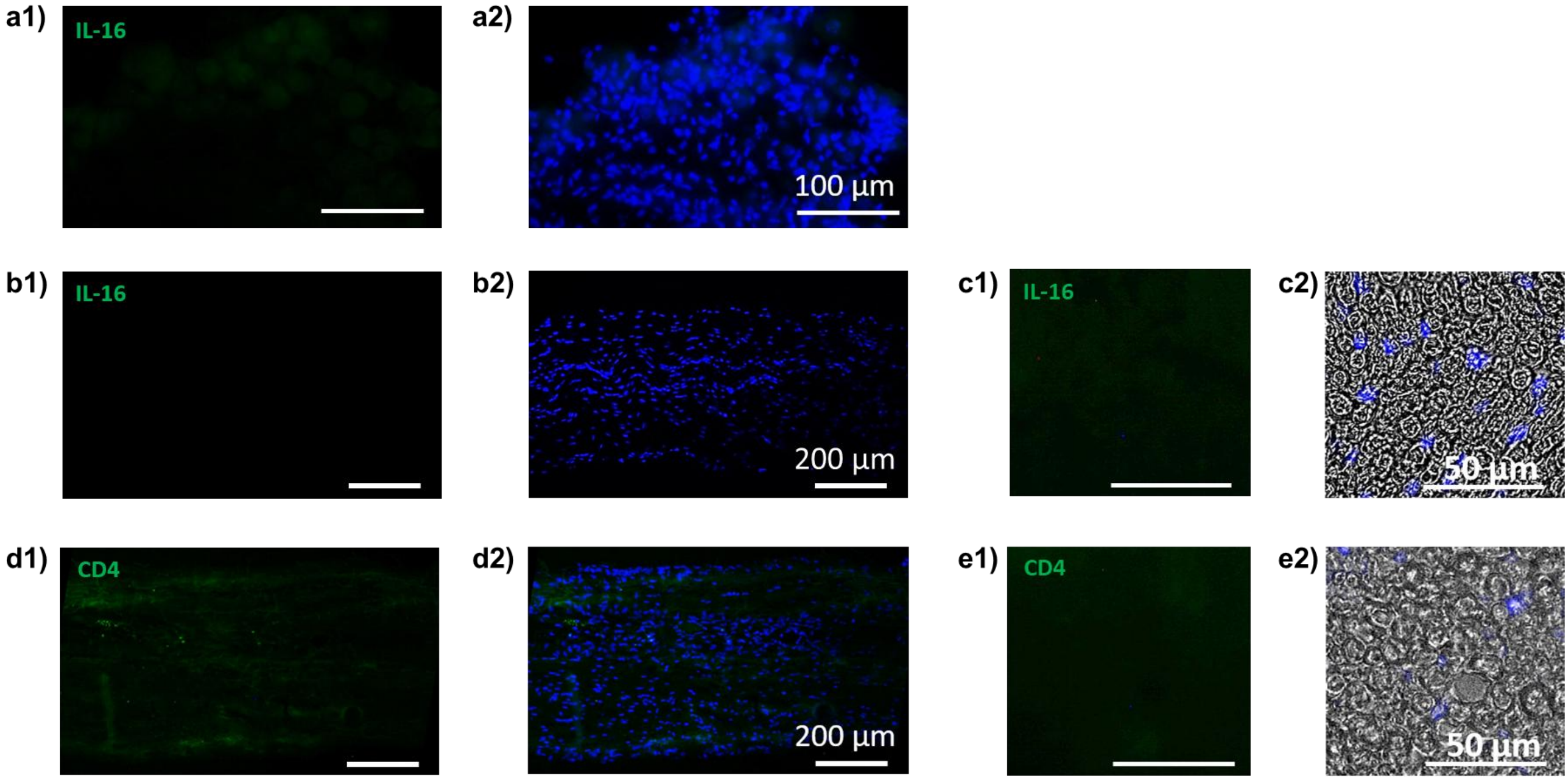

Supplementary Figure 1. Negative controls for IL-16 and CD4 immunofluorescence in dorsal root ganglia (DRG) and sciatic nerve. Representative fluorescence microscopy images of negative controls processed without the corresponding primary antibody against IL-16 or CD4. All images were acquired using a fluorescence microscope at the same magnification and exposure time as the corresponding positively stained samples. a1) Background fluorescence in the green channel used for IL-16 detection in a DRG. a2) Merged image of the green and blue (DAPI) nuclear staining channels in the DRG. b1) Background fluorescence in the green channel used for IL-16 detection in a longitudinal section of the sciatic nerve. b2) Merged image of the green and blue (DAPI) channels in a longitudinal section of the sciatic nerve. c1) Background fluorescence in the green channel used for IL-16 detection in a transverse section of the sciatic nerve. c2) Merged image of the green and blue (DAPI) channels and the corresponding bright-field image in a transverse section of the sciatic nerve. d1) Background fluorescence in the green channel used for CD4 detection in a longitudinal section of the sciatic nerve. d2) Merged image of the green and blue (DAPI) channels in a longitudinal section of the sciatic nerve. e1) Background fluorescence in the green channel used for CD4 detection in a transverse section of the sciatic nerve. e2) Merged image of the green and blue (DAPI) channels and the corresponding bright-field image in a transverse section of the sciatic nerve.

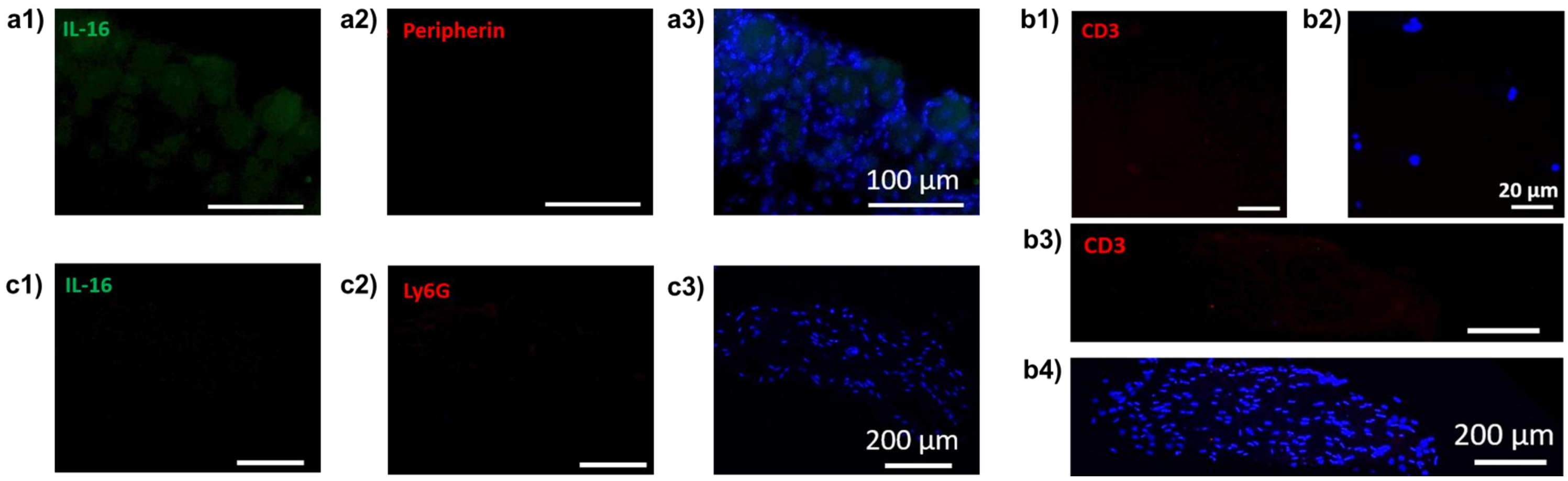

Supplementary Figure 2. Negative controls for IL-16, peripherin, immunofluorescence in dorsal root ganglia (DRG), CD3 and Ly6G, in sciatic nerve and thymus. Representative fluorescence microscopy images of negative controls processed without the corresponding primary antibodies against IL-16, peripherin, CD3 or Ly6G. Peripherin was used as a marker of small-diameter sensory neurons, CD3 as a marker of T lymphocytes and Ly6G as a marker of neutrophils. All images were acquired using a fluorescence microscope at the same magnification and exposure time as the corresponding positively stained samples. a1) Background fluorescence in the green channel used for IL-16 detection in a DRG. a2) Background fluorescence in the red channel used for peripherin detection in the DRG. a3) Merged image of the green, red and blue (DAPI) nuclear staining channels in the DRG. b1) Background fluorescence in the red channel used for CD3 detection in a thymus section. b2) Merged image of the red and blue (DAPI) channels in the thymus section. b3) Background fluorescence in the red channel used for CD3 detection in a longitudinal section of the sciatic nerve. b4) Merged image of the red and blue (DAPI) channels in a longitudinal section of sciatic nerve. c1) Background fluorescence in the green channel used for IL-16 detection in a longitudinal section of the sciatic nerve. c2) Background fluorescence in the red channel used for Ly6G detection in the longitudinal section of the sciatic nerve. c3) Merged image of the green, red and blue (DAPI) channels in the longitudinal section of the sciatic nerve.
